# Supplementary material for: Genetic architecture distinguishes tinnitus from hearing loss
Source: Nat Commun. 2024 Jan 19;15:614. doi: 10.1038/s41467-024-44842-x (PMC10799010; doi:10.1038/s41467-024-44842-x)
Supplement: Supplementary file 3 — Description of Additional Supplementary Files [file 41467_2024_44842_MOESM3_ESM.pdf]

Description of Additional Supplementary Files

File name: Supplementary Data 1
Description: Demographics of the study cohorts

File name: Supplementary Data 2
Description: Genome-wide significant loci associated with tinnitus in the European ancestry MVP, UKB, and GWAS meta-analysis, including 8,043,382 imputed SNPs present in both GWAS

File name: Supplementary Data 3
Description: Chromosome 8p23.1 inversion analyses in subjects of European ancestry

File name: Supplementary Data 4
Description: Functional mapping and annotation of the tinnitus GWAS meta-analysis in European ancestry participants, using the FUMA pipeline, and Polyfun+SUSIE for fine-mapping

File name: Supplementary Data 5
Description: A, Genome-wide significant loci associated with tinnitus in the MVP Hispanic (LAT) sample; B, Functional mapping and annotation of the tinnitus GWAS in MVP Hispanics (LAT)

File name: Supplementary Data 6
Description: Genome-wide significant loci associated with tinnitus in a cross-ancestry GWAS meta-analysis of European (EA), Hispanic (LAT), and African (AA) ancestries from UKB and MVP

File name: Supplementary Data 7
Description: Functional mapping and annotation of the cross-ancestry GWAS meta-analysis, using the FUMA pipeline, and Polyfun+SUSIE for fine-mapping

File name: Supplementary Data 8
Description: Polygenic risk score analysis (PRS) of tinnitus across different training and target populations

File name: Supplementary Data 9
Description: Significant genes in gene-based association analyses of tinnitus (European ancestry)

File name: Supplementary Data 10
Description: MAGMA Tissue-expression analyses (based on the European ancestry gene-based meta-analysis)

File name: Supplementary Data 11
Description: Cell type enrichment analyses in cochlear cells and the organ of Corti based on mouse gene expression data

File name: Supplementary Data 12
Description: MAGMA gene-set analysis, including 15,485 pre-defined curated gene sets and GO terms obtained from MsigDB

File name: Supplementary Data 13
Description: Results of drug-class enrichment analyses. Each class represents the combined result of all drugs combined according to sections of their Anatomical Therapeutic Chemical code

File name: Supplementary Data 14
Description: Drug-set enrichment analyses for nominally significant drug-sets. Each set represents all genes targeted by that drug.

File name: Supplementary Data 15
Description: Genetic architecture of tinnitus and comparison with hearing difficulty (HD) across the MVP and UKB cohorts

File name: Supplementary Data 16
Description: Identification of tinnitus-specific risk loci (based on the European ancestry tinnitus meta-analysis: main analysis, hearing adjusted, and CC GWAS)

File name: Supplementary Data 17
Description: Catalog of the pleiotropic effects of leading variants in risk loci identified across all analyses

File name: Supplementary Data 18
Description: Genetic Correlations of tinnitus with a broad range of traits and disorders using publicly available data (LDSC based on the MVP and UKB EA meta-analysis)

File name: Supplementary Data 19
Description: Datasets included in genomic structural equation modeling (gSEM) analysis for tinnitus, hearing difficulty, and psychiatric and health-related traits

File name: Supplementary Data 20
Description: gSEM confirmatory model fit for tinnitus and hearing difficulty

File name: Supplementary Data 21
Description: Regional association plots for tinnitus risk loci in the (a) European, (b) Hispanic, (c) African, and (d) cross-ancestry GWAS
