# Supplementary material for: Genetic architecture distinguishes tinnitus from hearing loss
Source: Nat Commun. 2024 Jan 19;15:614. doi: 10.1038/s41467-024-44842-x (PMC10799010; doi:10.1038/s41467-024-44842-x)
Supplement: Supplementary file 24 — Supplementary Data 21 [file 41467_2024_44842_MOESM24_ESM.pdf]

Supplementary Data 21: Regional association plots for tinnitus risk loci in the (a) European, (b) Hispanic, (c) African, and (d) cross-ancestry GWAS. The x-axis represents the base pair (hg19) genetic position of SNPs. The y-axis represents $-\log_{10}$ p-values of SNP association with tinnitus. LD estimates of surrounding SNPs with the labeled index SNP is indicated by color (color bar on side of plot indicates color coding of r^2 values). LD r^2 values estimated based on 1KGPp3 data, where EUR samples were used as reference genotypes for European ancestry, AMR used for Hispanic ancestry, and AFR used for African ancestry. Local estimates of recombination rate are indicated in light blue (legend on vertical axis at right). Gene names, strands, and boundaries are shown in the box below the regional plot. Locus number is indicated on the bottom left of each figure. Where the leading SNP on a given locus varied between European and cross-ancestry GWAS, both rs-ids are noted and LD is plotted in reference to the European ancestry rs-id.

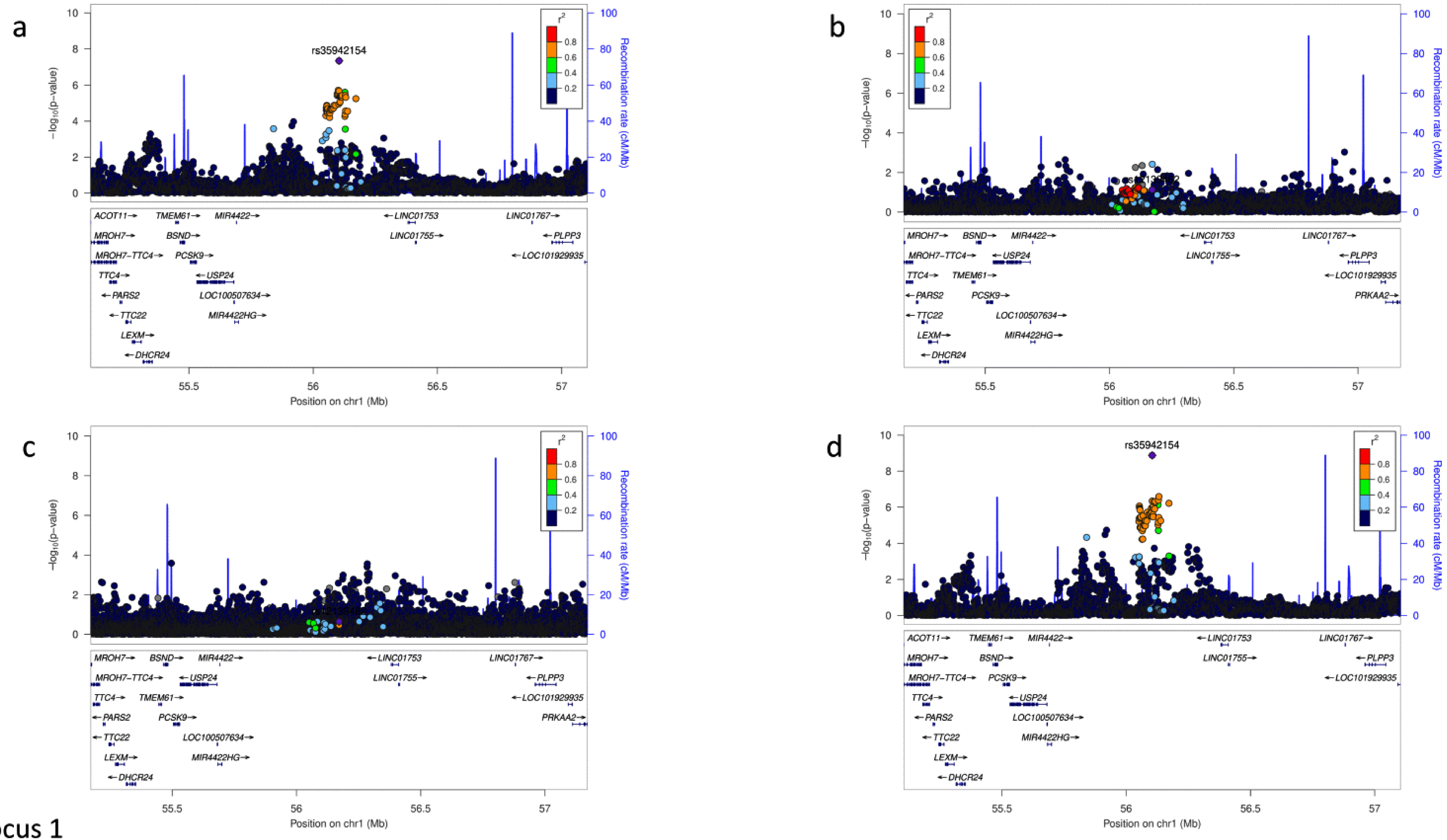
a
b
c
d
rs35942154
rs35942154
Position on chr1 (Mb)
Position on chr1 (Mb)
Position on chr1 (Mb)
Position on chr1 (Mb)
Recombination rate (cM/Mb)
r²
ACOT11
MROH7
MROH7-TTC4
TTC4
PARS2
TTC22
LEXM
DHCR24
TMEM61
BSND
PCSK9
USP24
LOC100507634
MIR4422
MIR4422HG
LINC01753
LINC01755
LINC01767
PLPP3
LOC101929935
PRKAA2

Locus 1

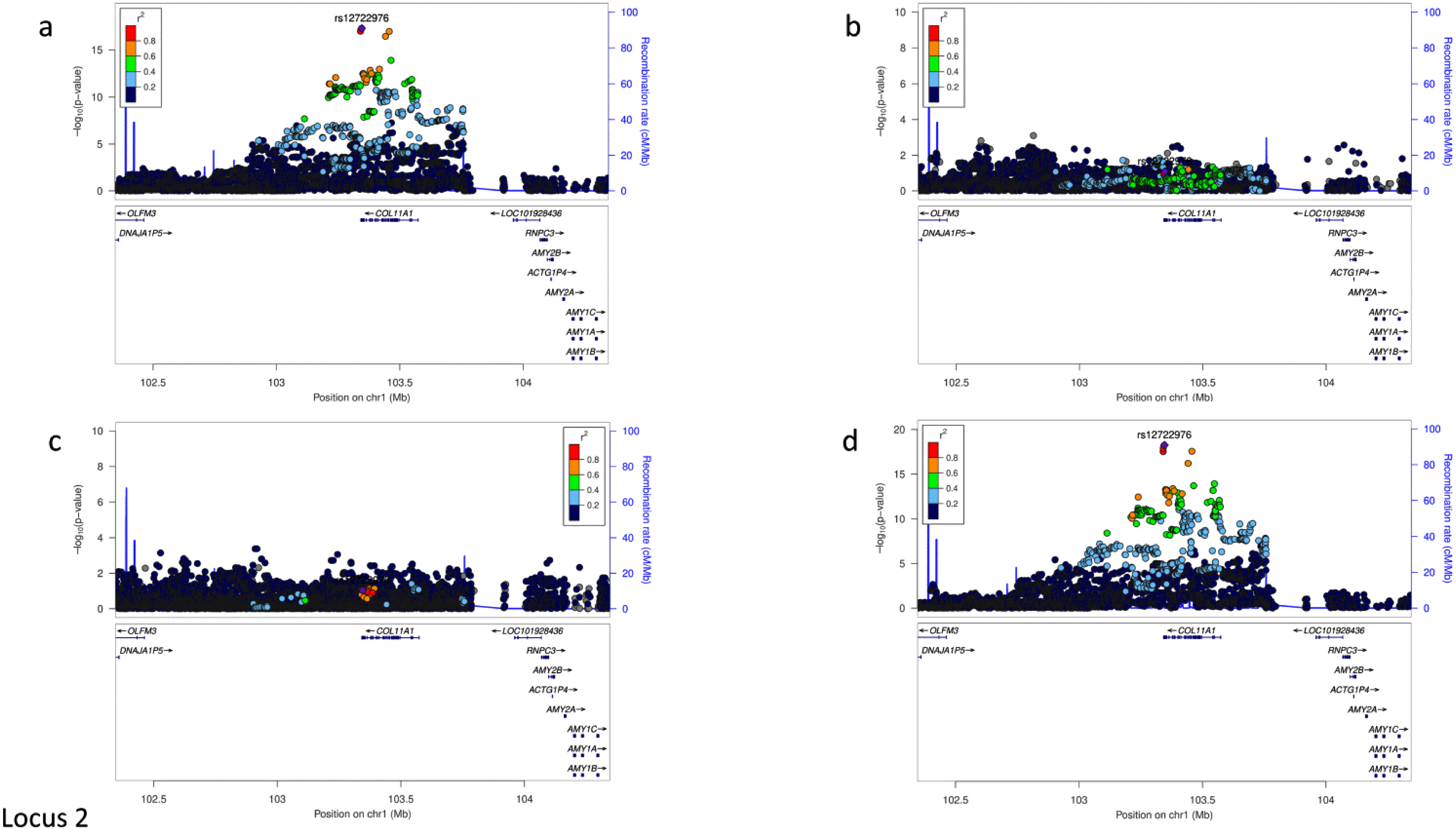

a
b
c
d
rs12722976
r2
−log10(p−value)
Recombination rate (cM/Mb)
Position on chr1 (Mb)
OLFM3
DNAJA1P5
COL11A1
LOC101928436
RNPC3
AMY2B
ACTG1P4
AMY2A
AMY1C
AMY1A
AMY1B

Locus 2

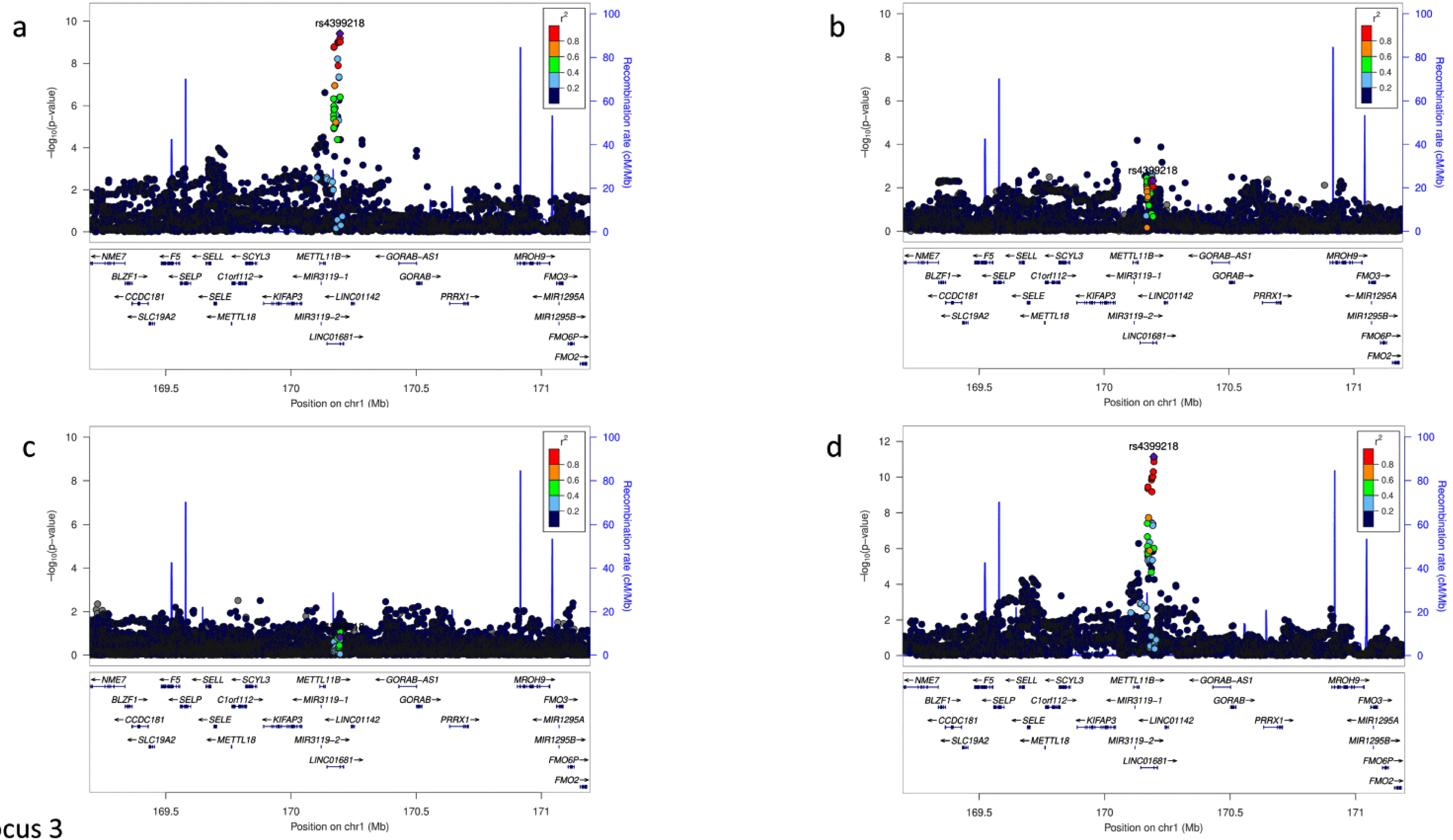
a
b
c
d
rs4399218
−log10(p−value)
Recombination rate (cM/Mb)
r2
Position on chr1 (Mb)
169.5
170
170.5
171
NME7
BLZF1
CCDC181
SLC19A2
F5
SELP
SELL
SELE
METTL18
SCYL3
C1orf112
KIFAP3
METTL11B
MIR3119−1
LINC01142
MIR3119−2
LINC01681
GORAB−AS1
GORAB
PRRX1
MROH9
FMO3
MIR1295A
MIR1295B
FMO6P
FMO2

Locus 3

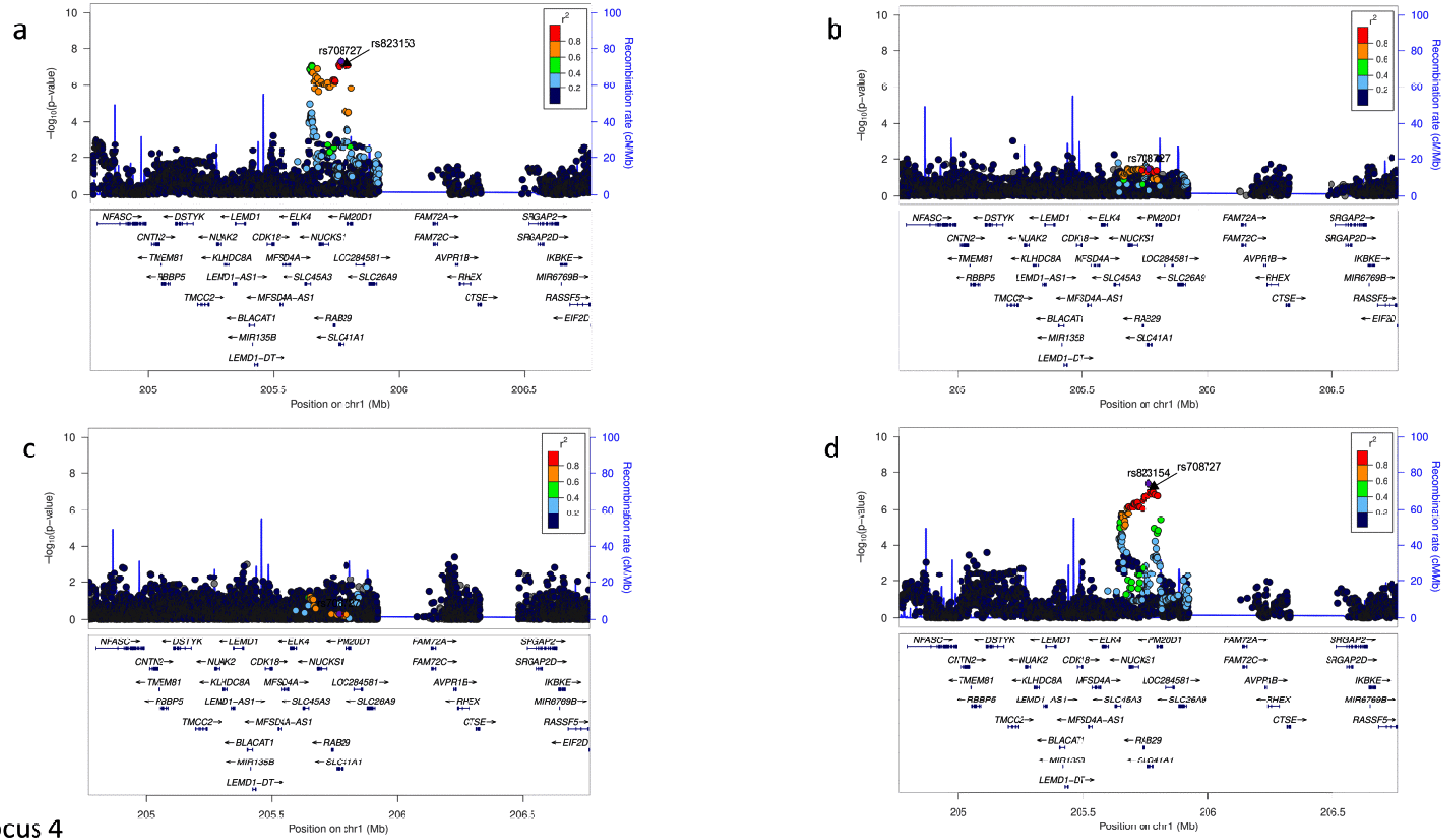
a
b
c
d
rs708727
rs823153
rs823154
−log10(p−value)
Recombination rate (cM/Mb)
r2
0.8
0.6
0.4
0.2
Position on chr1 (Mb)
205
205.5
206
206.5
NFASC
DSTYK
LEMD1
ELK4
PM20D1
FAM72A
SRGAP2
CNTN2
NUAK2
CDK18
NUCKS1
FAM72C
SRGAP2D
TMEM81
KLHDC8A
MFSD4A
LOC284581
AVPR1B
IKBKE
RBBP5
LEMD1-AS1
SLC45A3
SLC26A9
RHEX
MIR6769B
TMCC2
MFSD4A-AS1
CTSE
RASSF5
BLACAT1
RAB29
EIF2D
MIR135B
SLC41A1
LEMD1-DT

Locus 4

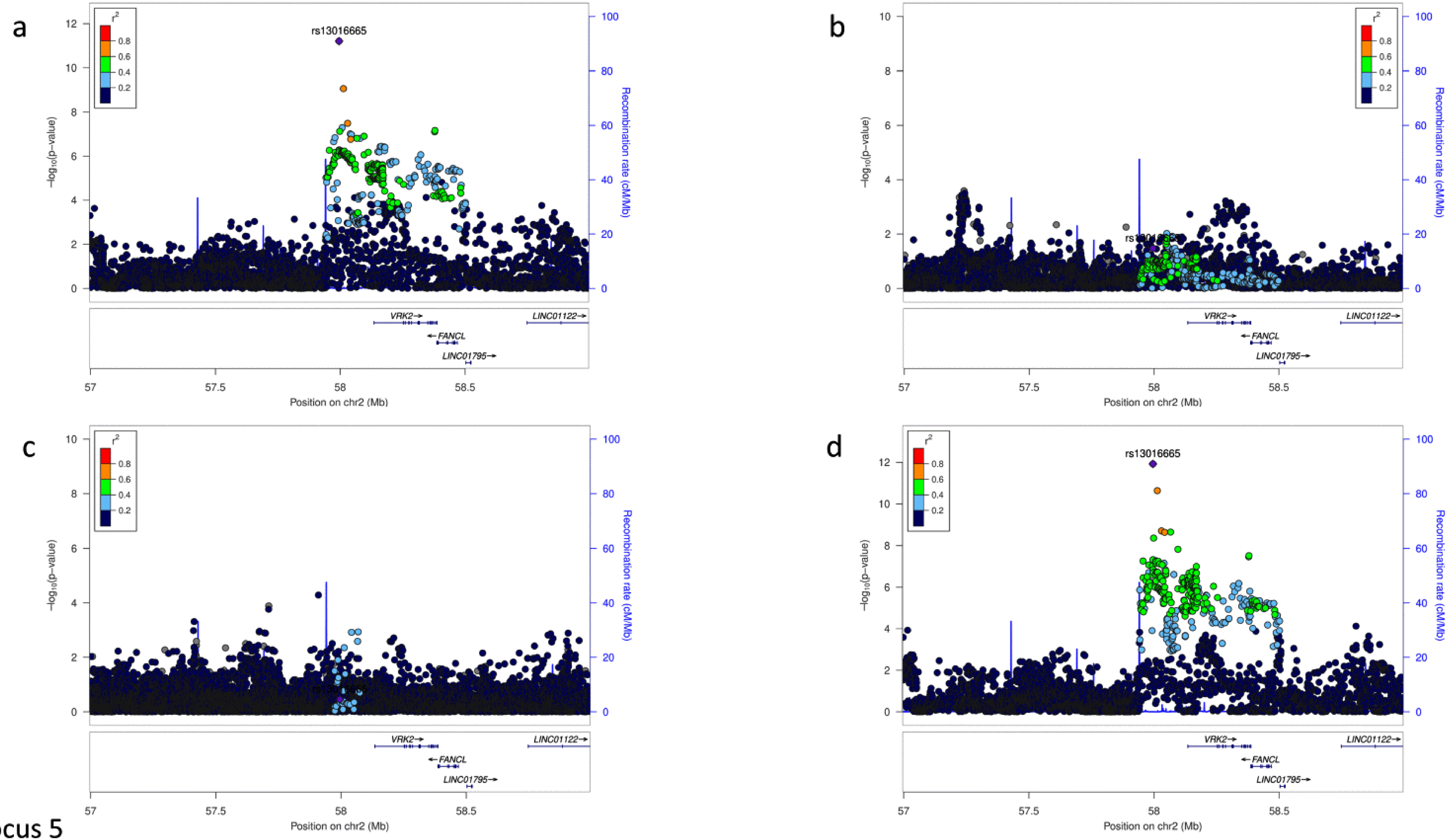

a
b
c
d
rs13016665
r2
0.8
0.6
0.4
0.2
−log10(p−value)
Recombination rate (cM/Mb)
VRK2→
←FANCL
LINC01795→
LINC01122→
57
57.5
58
58.5
Position on chr2 (Mb)

Locus 5

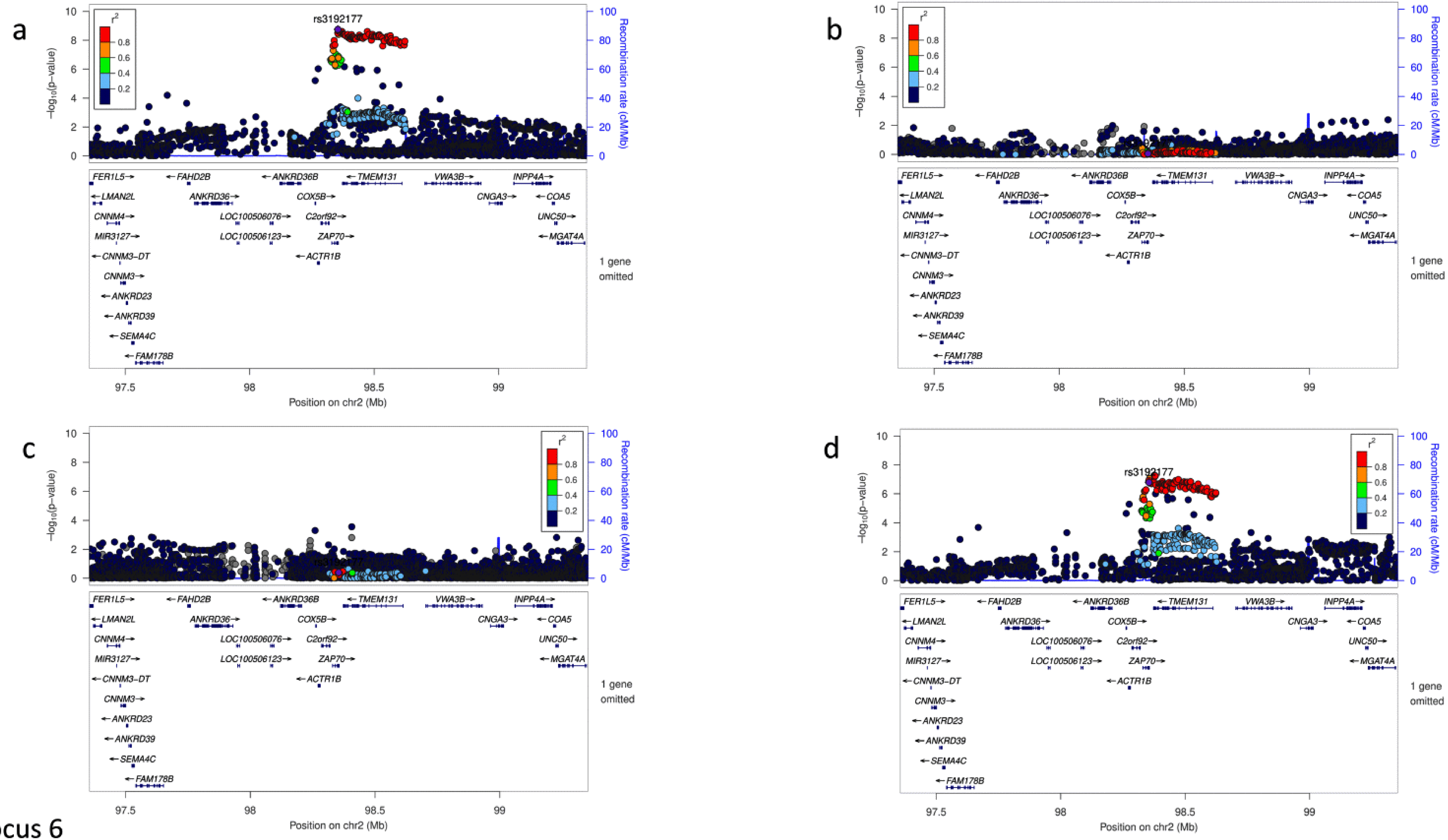
a
b
c
d
rs3192177
r2
0.8
0.6
0.4
0.2
−log10(p−value)
Recombination rate (cM/Mb)
FER1L5→
←LMAN2L
CNNM4→
MIR3127→
←CNNM3−DT
CNNM3→
←ANKRD23
←ANKRD39
←SEMA4C
←FAM178B
←FAHD2B
ANKRD36→
←ANKRD36B
LOC100506076→
LOC100506123→
COX5B→
C2orf92→
ZAP70→
←ACTR1B
←TMEM131
VWA3B→
CNGA3→
INPP4A→
←COA5
UNC50→
←MGAT4A
1 gene omitted
97.5
98
98.5
99
Position on chr2 (Mb)

Locus 6

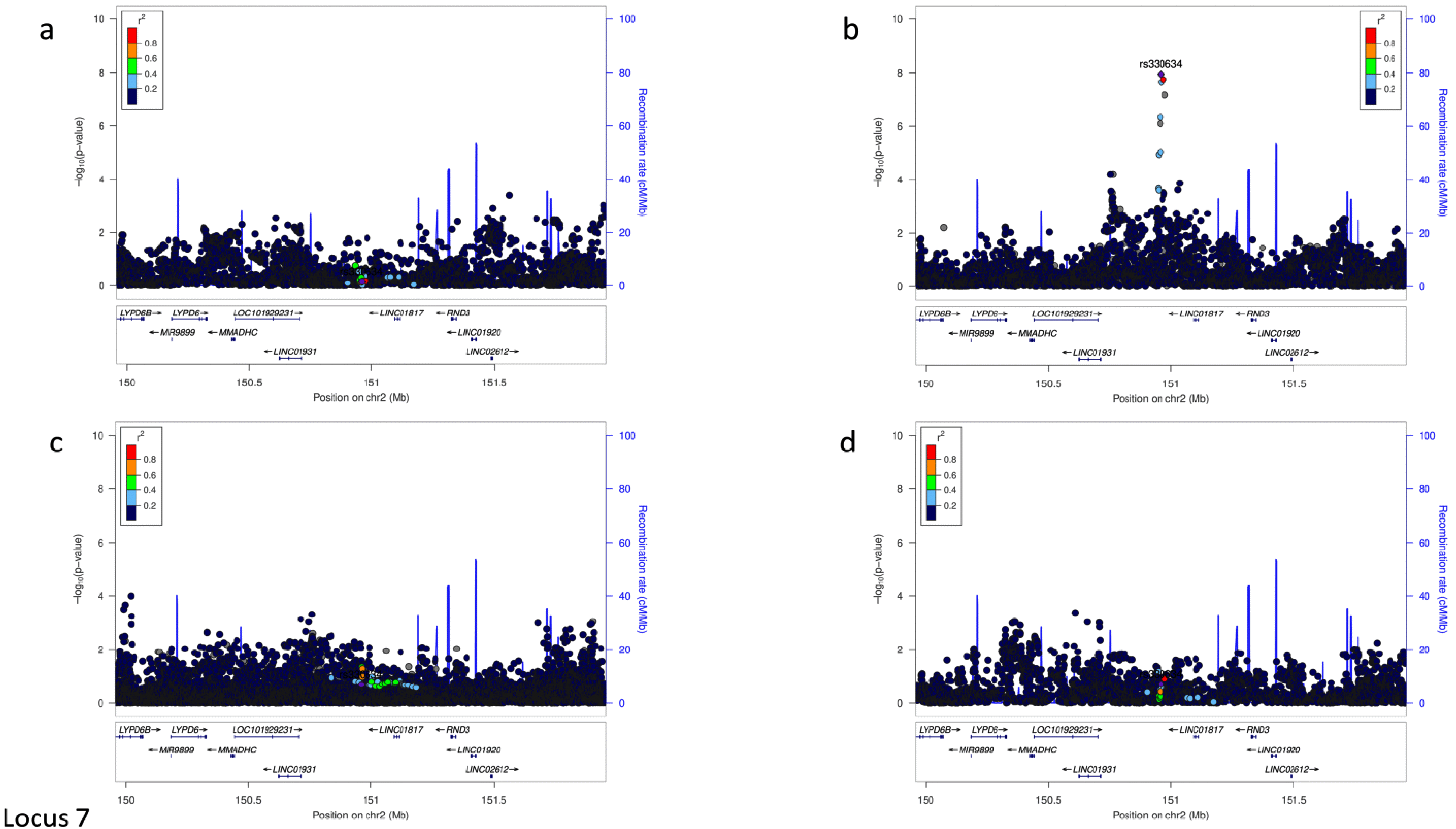
a
b
c
d
rs330634
r²
0.8
0.6
0.4
0.2
−log10(p−value)
Recombination rate (cM/Mb)
LYPD6B
LYPD6
LOC101929231
LINC01817
RND3
MIR9899
MMADHC
LINC01920
LINC01931
LINC02612
Position on chr2 (Mb)

Locus 7

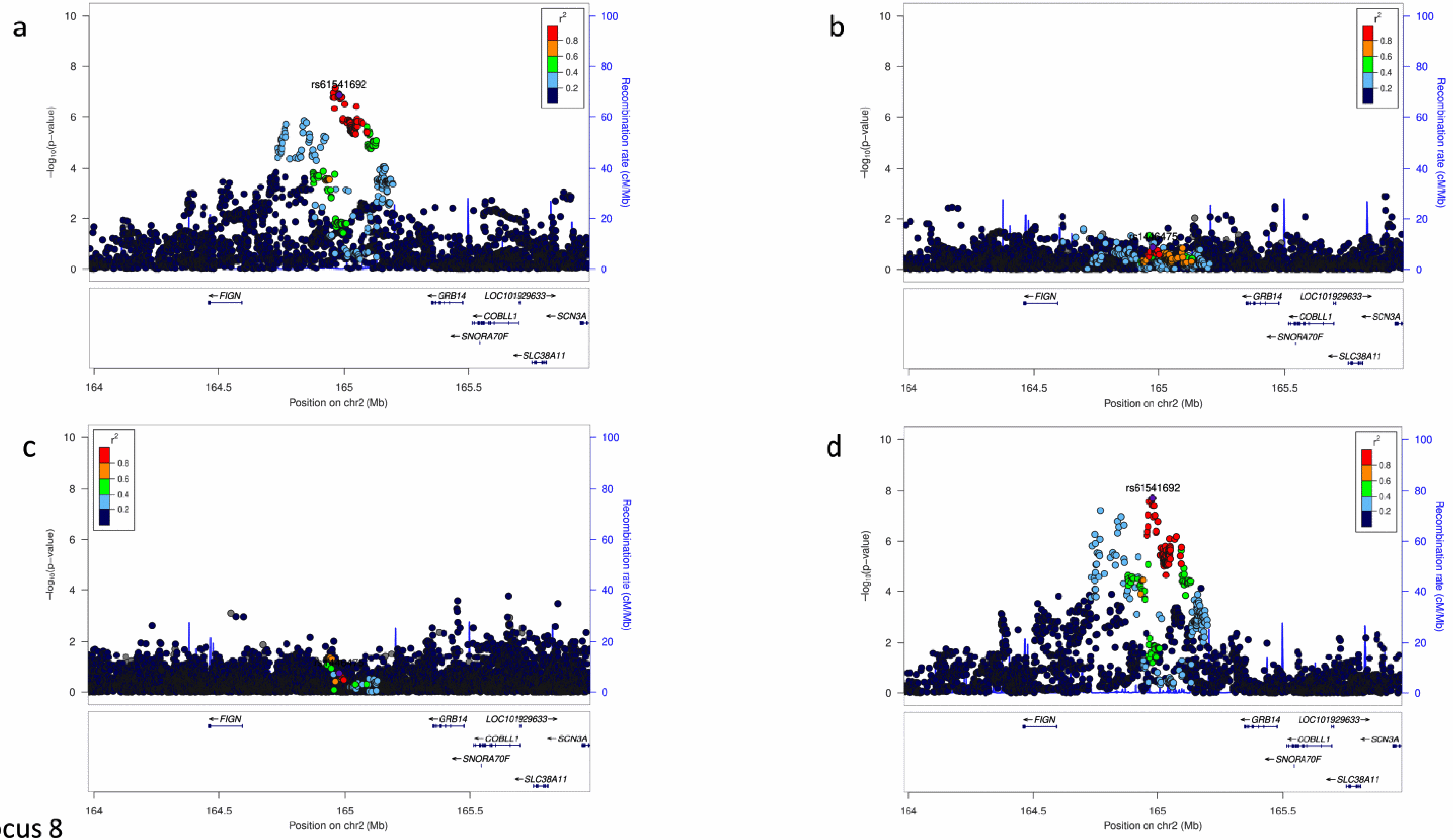
a
b
c
d
rs61541692
r2
0.8
0.6
0.4
0.2
−log10(p−value)
Recombination rate (cM/Mb)
FIGN
GRB14
LOC101929633
COBLL1
SCN3A
SNORA70F
SLC38A11
164
164.5
165
165.5
Position on chr2 (Mb)

Locus 8

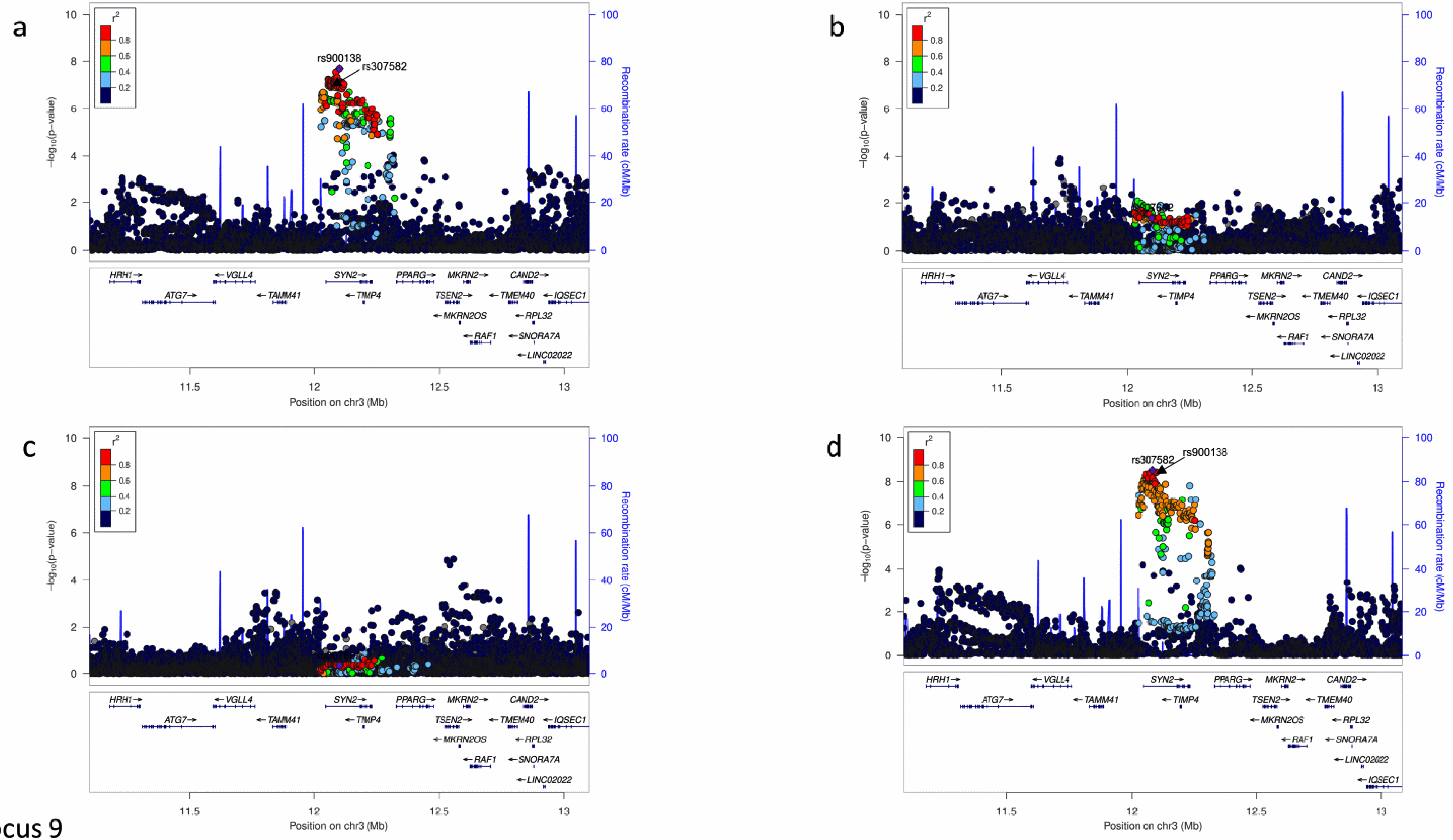
a
b
c
d
rs900138
rs307582
r2
0.8
0.6
0.4
0.2
−log10(p−value)
Recombination rate (cM/Mb)
HRH1
VGLL4
SYN2
PPARG
MKRN2
CAND2
ATG7
TAMM41
TIMP4
TSEN2
TMEM40
IQSEC1
MKRN2OS
RPL32
RAF1
SNORA7A
LINC02022
Position on chr3 (Mb)
11.5
12
12.5
13

Locus 9

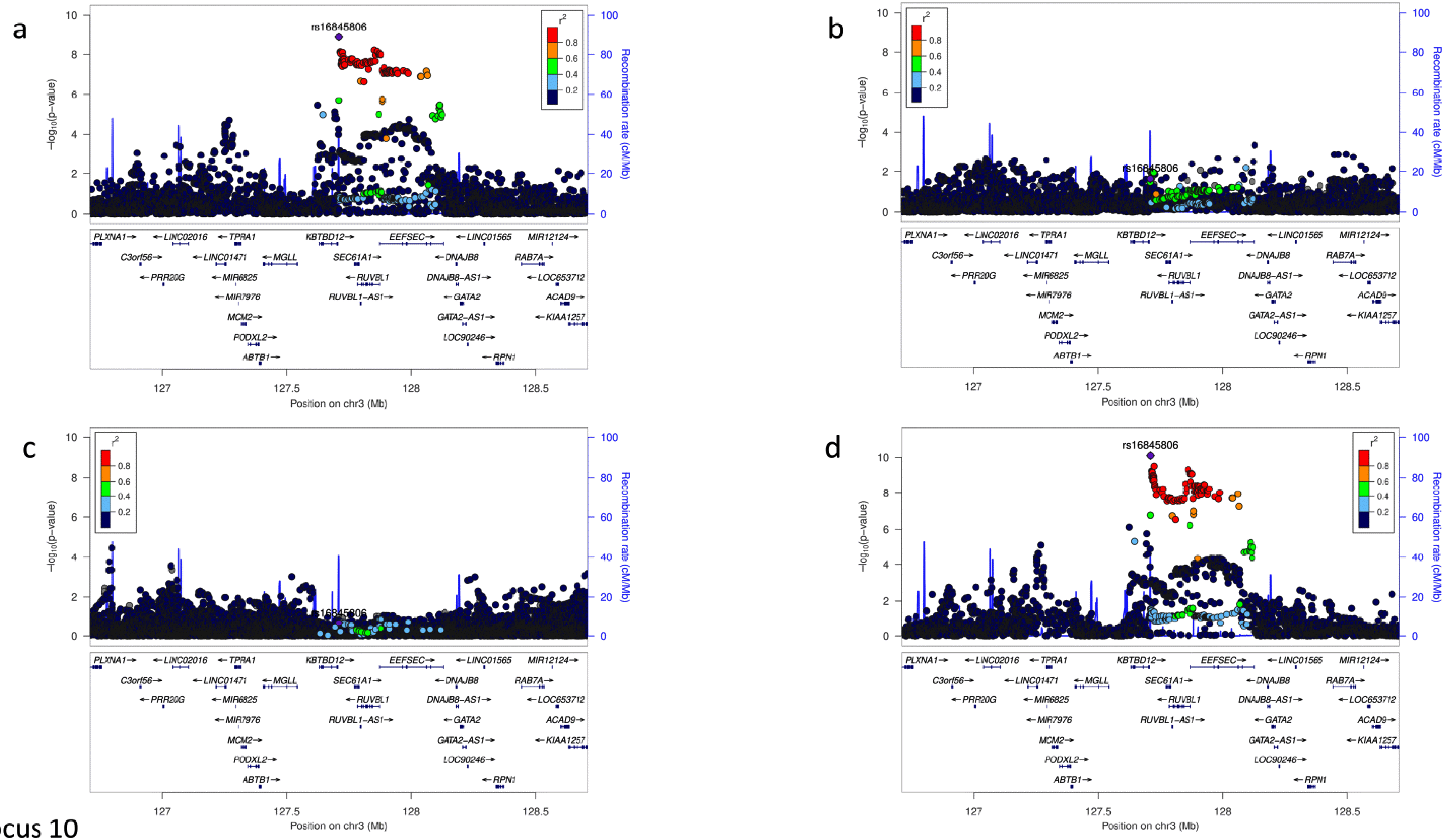
a
b
c
d
rs16845806
r²
0.8
0.6
0.4
0.2
−log10(p−value)
Recombination rate (cM/Mb)
Position on chr3 (Mb)
127
127.5
128
128.5
PLXNA1→
←LINC02016
←TPRA1
KBTBD12→
EEFSEC→
←LINC01565
MIR12124→
C3orf56→
←LINC01471
←MGLL
SEC61A1→
←DNAJB8
RAB7A→
←PRR20G
←MIR6825
←RUVBL1
DNAJB8−AS1→
←LOC653712
←MIR7976
RUVBL1−AS1→
←GATA2
ACAD9→
MCM2→
GATA2−AS1→
←KIAA1257
PODXL2→
LOC90246→
ABTB1→
←RPN1

Locus 10

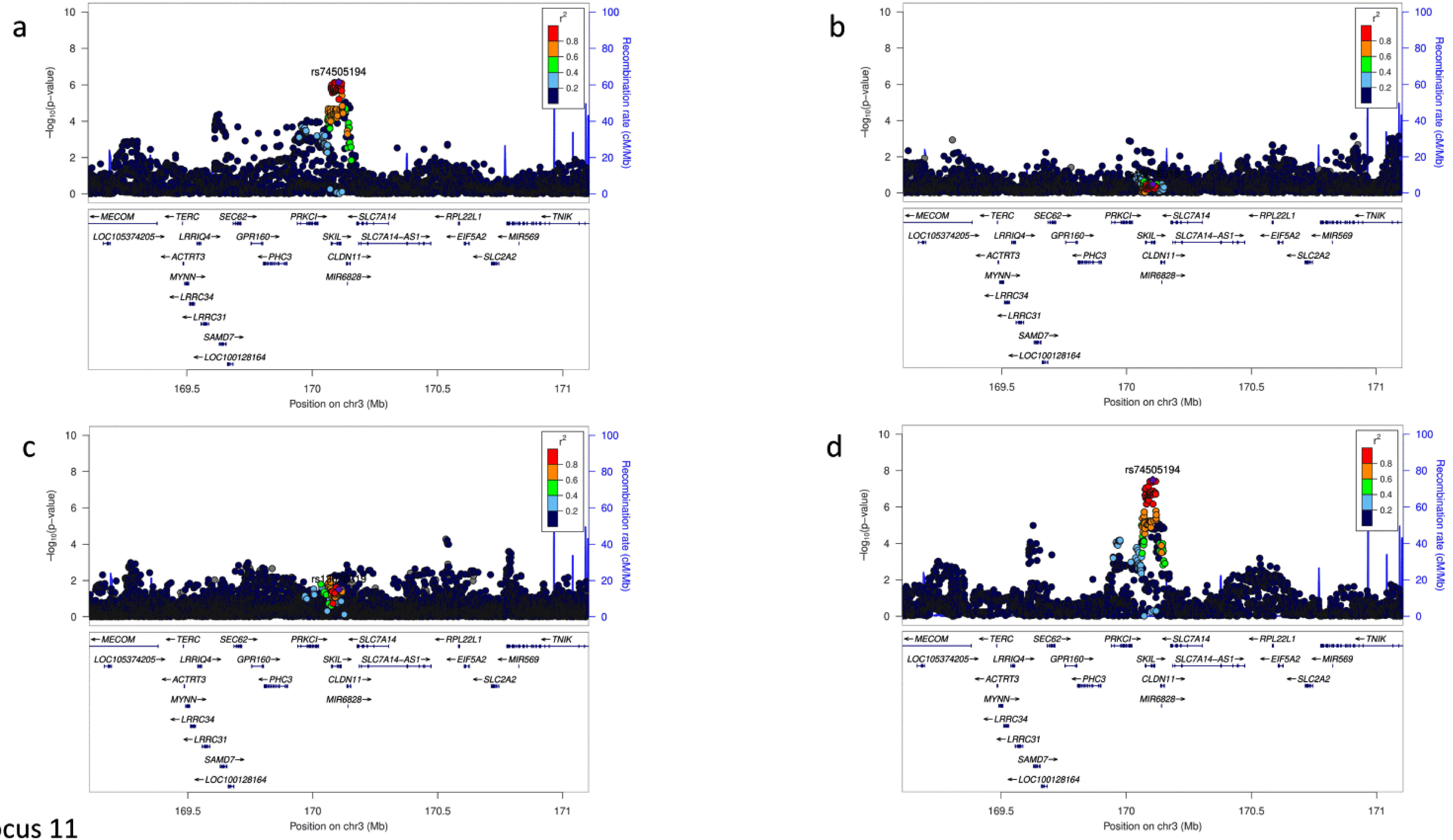
a
b
c
d
rs74505194
−log10(p−value)
Recombination rate (cM/Mb)
r2
0.8
0.6
0.4
0.2
MECOM
TERC
SEC62
PRKCI
SLC7A14
RPL22L1
TNIK
LOC105374205
LRRIQ4
GPR160
SKIL
SLC7A14−AS1
EIF5A2
MIR569
ACTRT3
PHC3
CLDN11
SLC2A2
MYNN
MIR6828
LRRC34
LRRC31
SAMD7
LOC100128164
169.5
170
170.5
171
Position on chr3 (Mb)

Locus 11

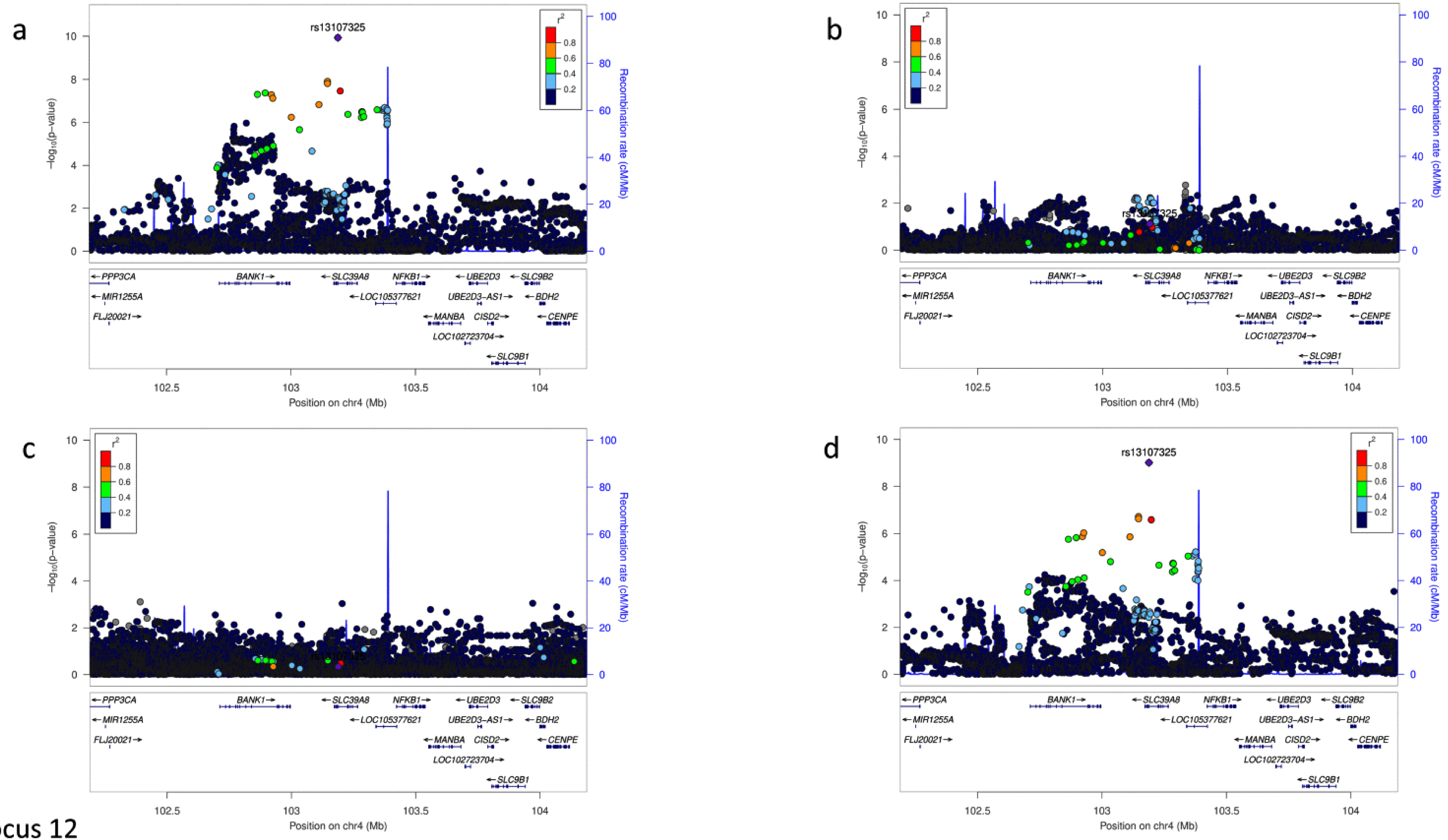
a
b
c
d
rs13107325
−log10(p−value)
Recombination rate (cM/Mb)
Position on chr4 (Mb)
r2
0.8
0.6
0.4
0.2
PPP3CA
MIR1255A
FLJ20021
BANK1
SLC39A8
LOC105377621
NFKB1
MANBA
UBE2D3
UBE2D3−AS1
CISD2
LOC102723704
SLC9B1
SLC9B2
BDH2
CENPE

Locus 12

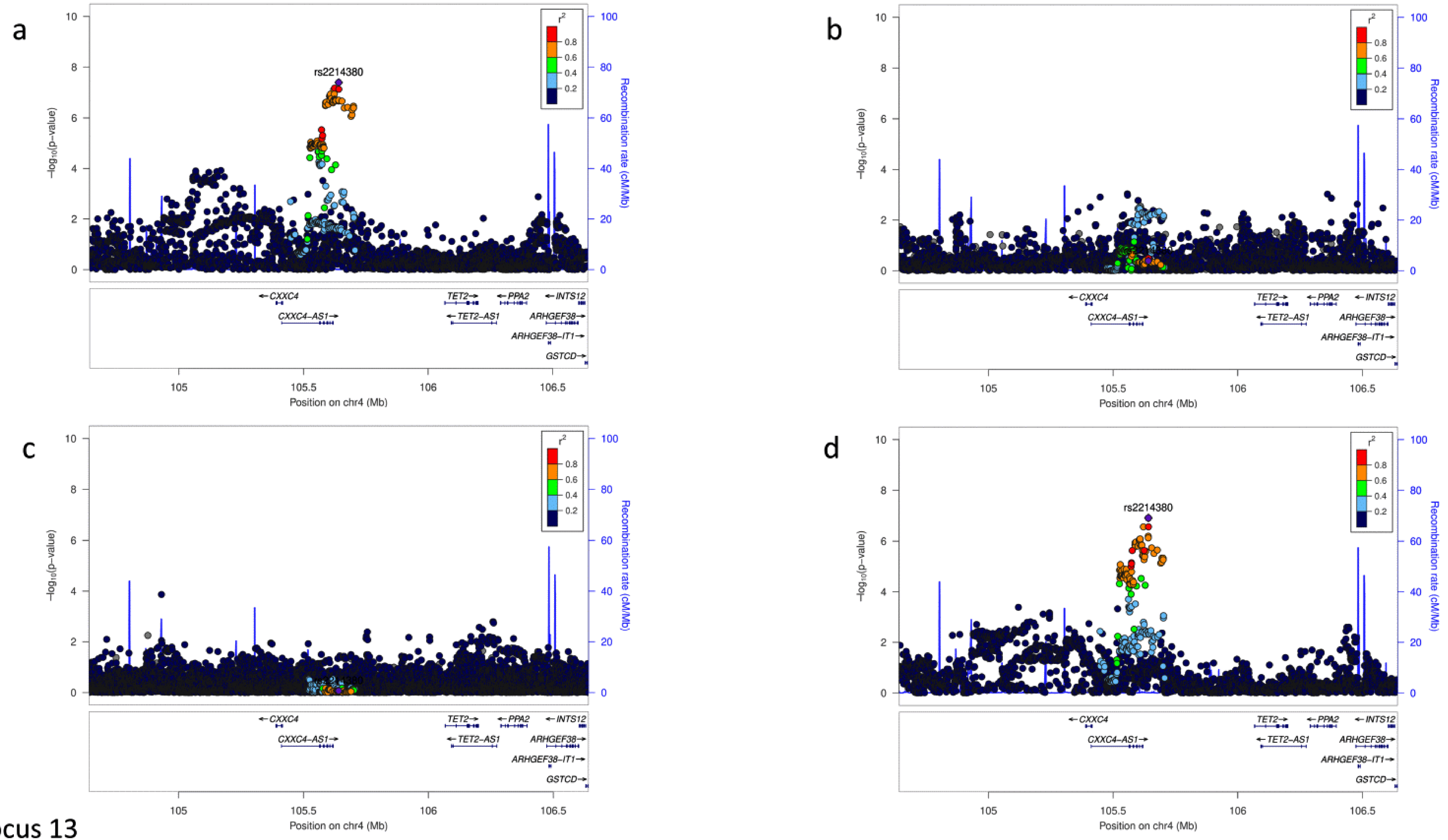
a
b
c
d
rs2214380
rs2214380
r²
0.8
0.6
0.4
0.2
−log10(p−value)
Recombination rate (cM/Mb)
Position on chr4 (Mb)
105
105.5
106
106.5
CXXC4
CXXC4−AS1
TET2
TET2−AS1
PPA2
INTS12
ARHGEF38
ARHGEF38−IT1
GSTCD

Locus 13

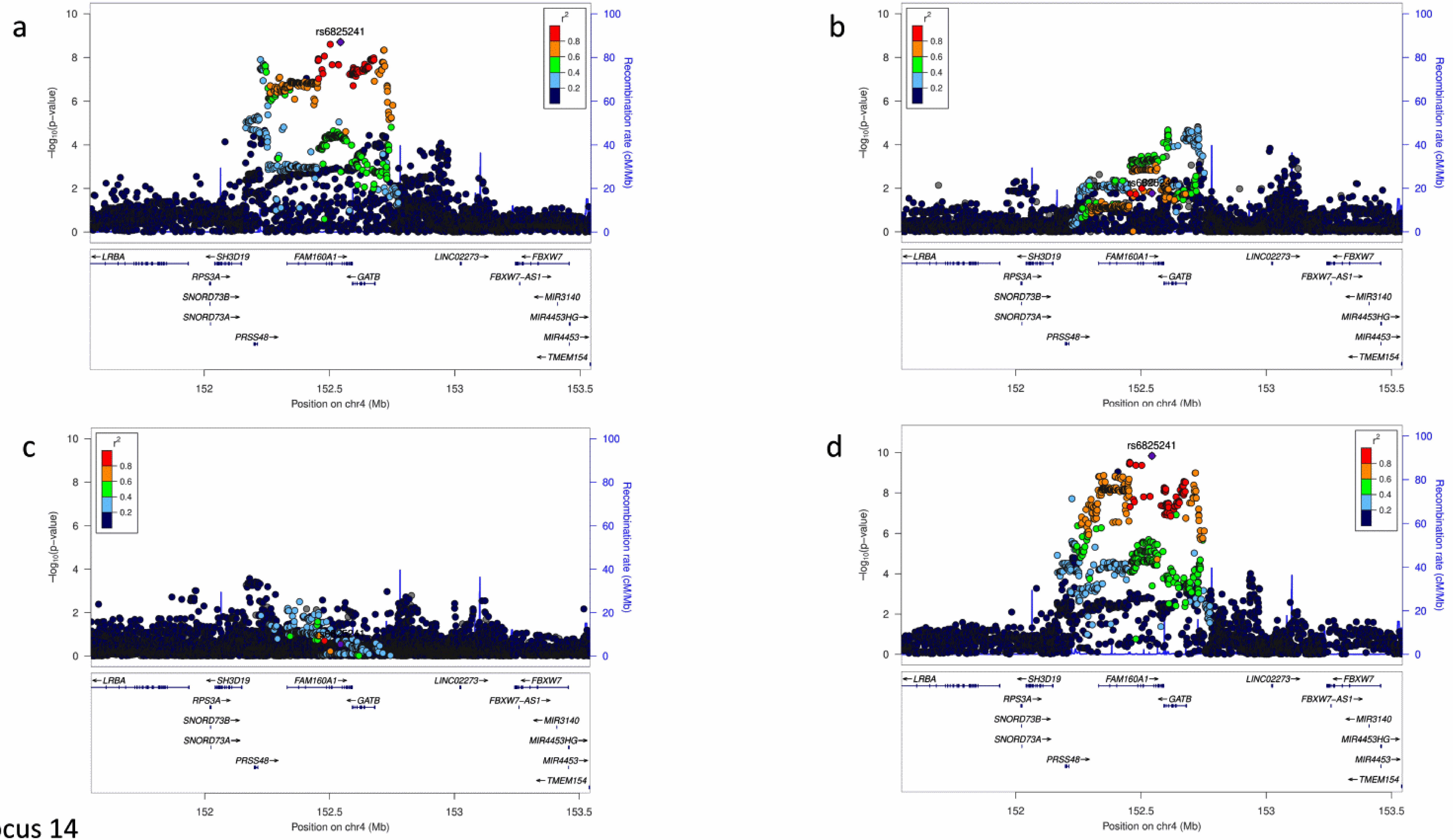
a
b
c
d
rs6825241
−log10(p−value)
Recombination rate (cM/Mb)
Position on chr4 (Mb)
r2
0.8
0.6
0.4
0.2
LRBA
SH3D19
RPS3A
SNORD73B
SNORD73A
PRSS48
FAM160A1
GATB
LINC02273
FBXW7
FBXW7−AS1
MIR3140
MIR4453HG
MIR4453
TMEM154
152
152.5
153
153.5

Locus 14

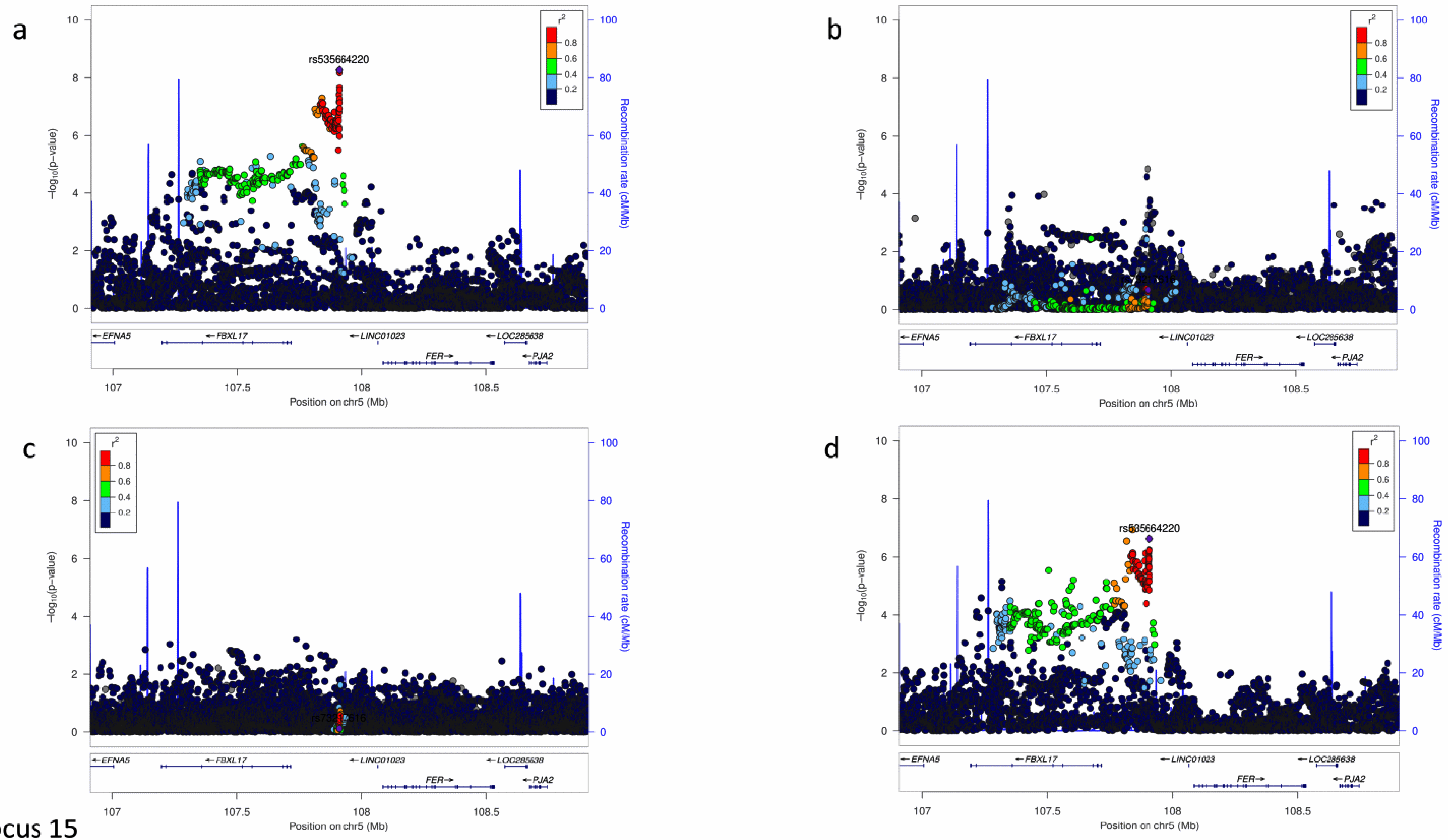
a
b
c
d
rs535664220
rs535664220
r²
0.8
0.6
0.4
0.2
−log10(p−value)
Recombination rate (cM/Mb)
Position on chr5 (Mb)
EFNA5
FBXL17
LINC01023
LOC285638
FER
PJA2
107
107.5
108
108.5

Locus 15

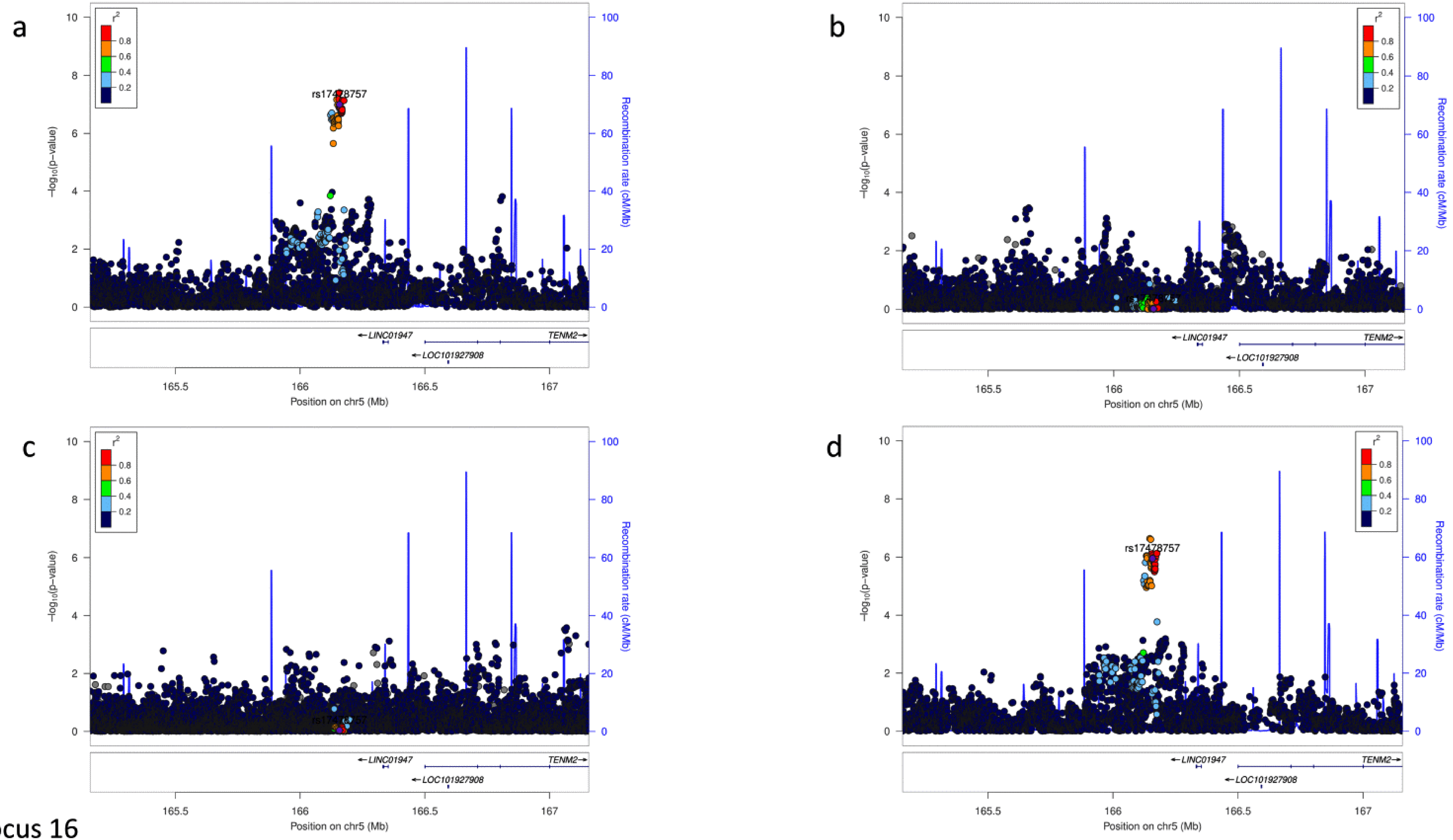

a
b
c
d
rs17478757
r2
0.8
0.6
0.4
0.2
−log10(p−value)
Recombination rate (cM/Mb)
LINC01947
LOC101927908
TENM2
165.5
166
166.5
167
Position on chr5 (Mb)

Locus 16

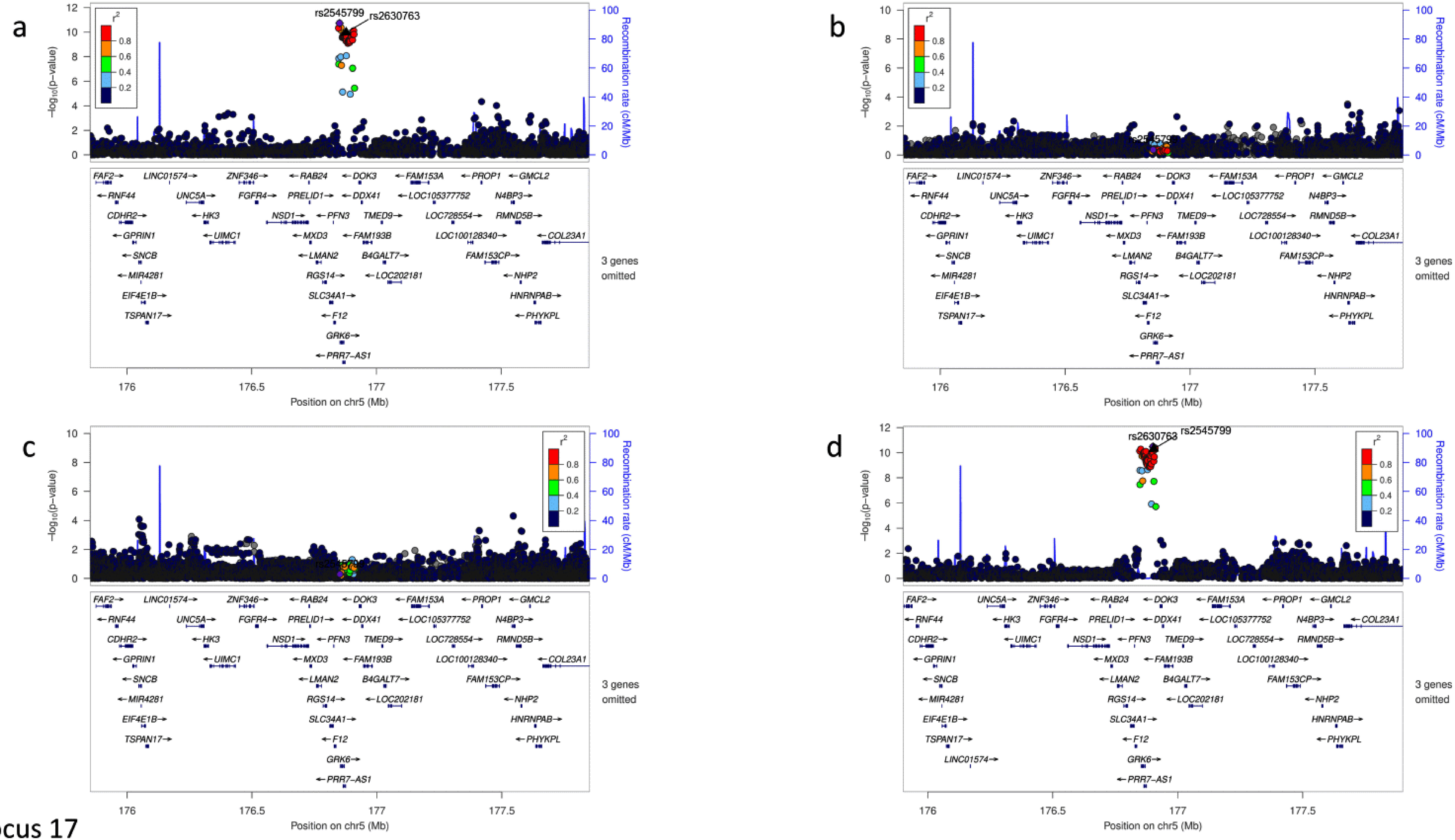
a
rs2545799
rs2630763
b
c
d
r²
0.8
0.6
0.4
0.2
−log10(p−value)
Recombination rate (cM/Mb)
Position on chr5 (Mb)
176
176.5
177
177.5
FAF2
RNF44
CDHR2
GPRIN1
SNCB
MIR4281
EIF4E1B
TSPAN17
LINC01574
UNC5A
HK3
UIMC1
ZNF346
FGFR4
NSD1
RAB24
PRELID1
MXD3
LMAN2
RGS14
SLC34A1
F12
GRK6
PRR7−AS1
PFN3
DOK3
DDX41
FAM193B
TMED9
B4GALT7
LOC202181
FAM153A
LOC105377752
LOC728554
LOC100128340
FAM153CP
PROP1
N4BP3
RMND5B
NHP2
HNRNPAB
PHYKPL
GMCL2
COL23A1
3 genes omitted

Locus 17

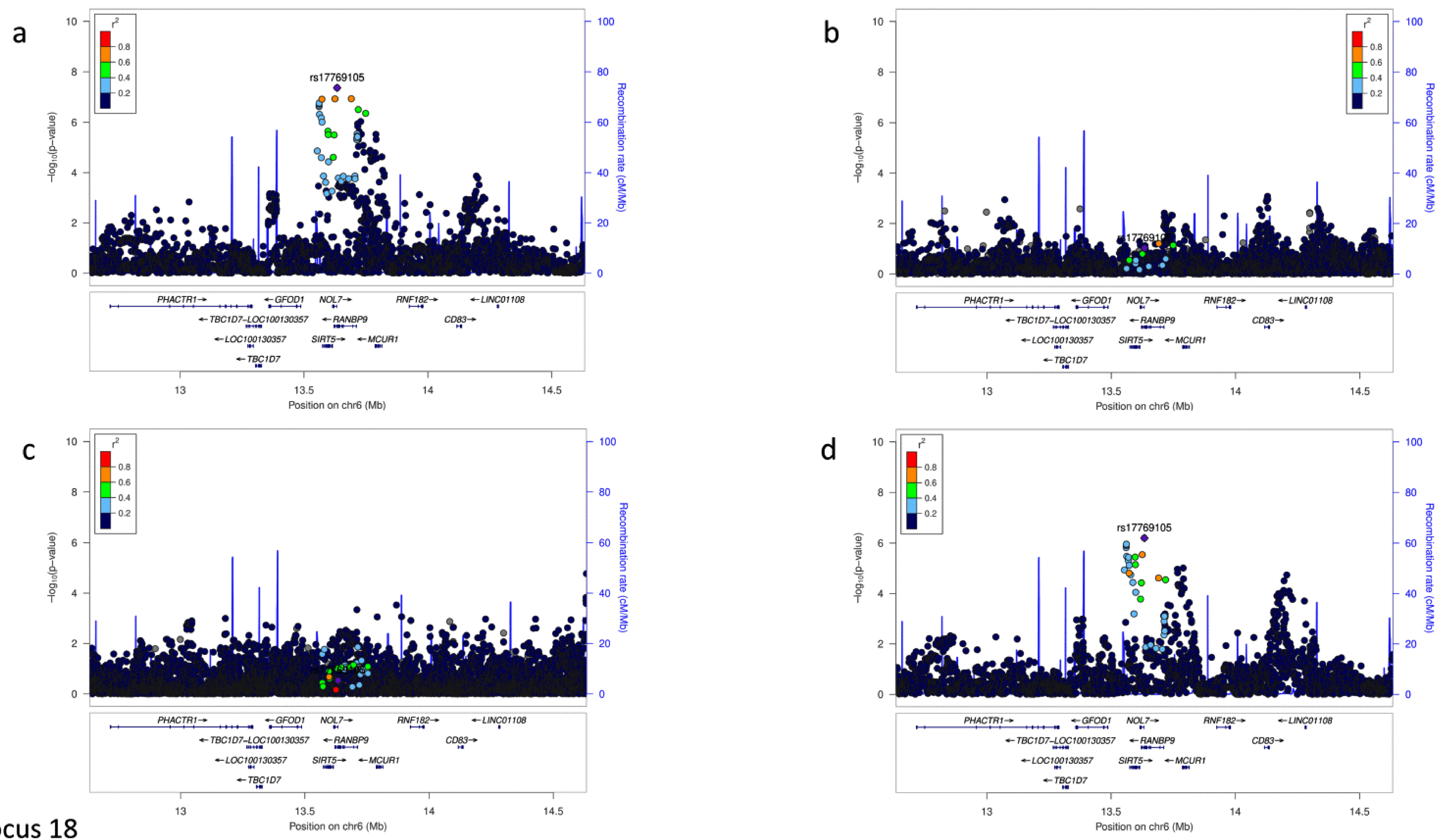
a
b
c
d
rs17769105
r2
0.8
0.6
0.4
0.2
−log10(p−value)
Recombination rate (cM/Mb)
PHACTR1→
←GFOD1
NOL7→
RNF182→
←LINC01108
←TBC1D7−LOC100130357
←RANBP9
CD83→
←LOC100130357
SIRT5→
←MCUR1
←TBC1D7
13
13.5
14
14.5
Position on chr6 (Mb)

Locus 18

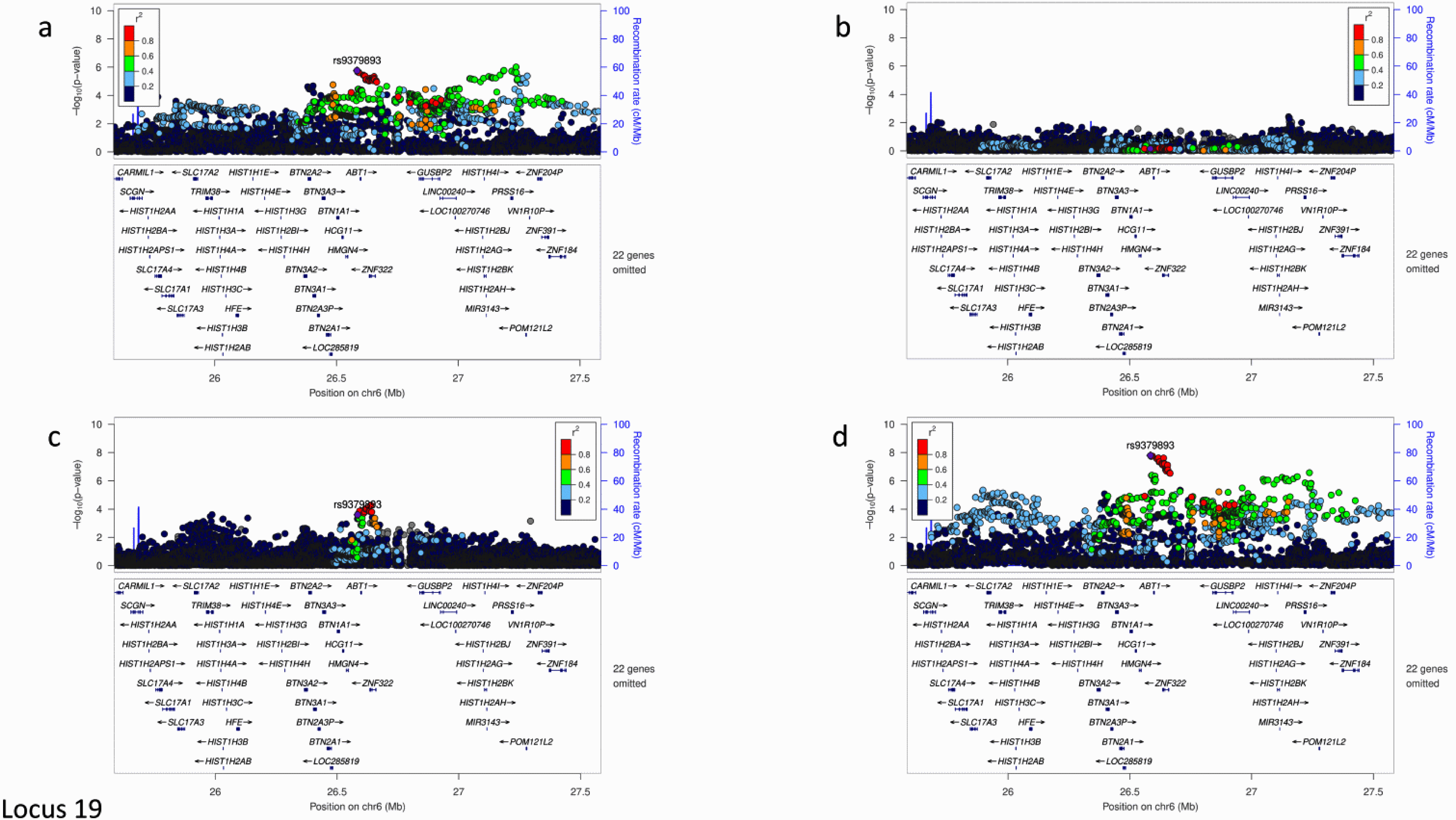
a
b
c
d
rs9379893
r²
0.8
0.6
0.4
0.2
−log10(p−value)
Recombination rate (cM/Mb)
Position on chr6 (Mb)
22 genes omitted
CARMIL1
SLC17A2
HIST1H1E
BTN2A2
ABT1
GUSBP2
HIST1H4I
ZNF204P
SCGN
TRIM38
HIST1H4E
BTN3A3
LINC00240
PRSS16
HIST1H2AA
HIST1H1A
HIST1H3G
BTN1A1
LOC100270746
VN1R10P
HIST1H2BA
HIST1H3A
HIST1H2BI
HCG11
HIST1H2BJ
ZNF391
HIST1H2APS1
HIST1H4A
HIST1H4H
HMGN4
HIST1H2AG
ZNF184
SLC17A4
HIST1H4B
BTN3A2
ZNF322
HIST1H2BK
SLC17A1
HIST1H3C
BTN3A1
HIST1H2AH
SLC17A3
HFE
BTN2A3P
MIR3143
HIST1H3B
BTN2A1
POM121L2
HIST1H2AB
LOC285819

Locus 19

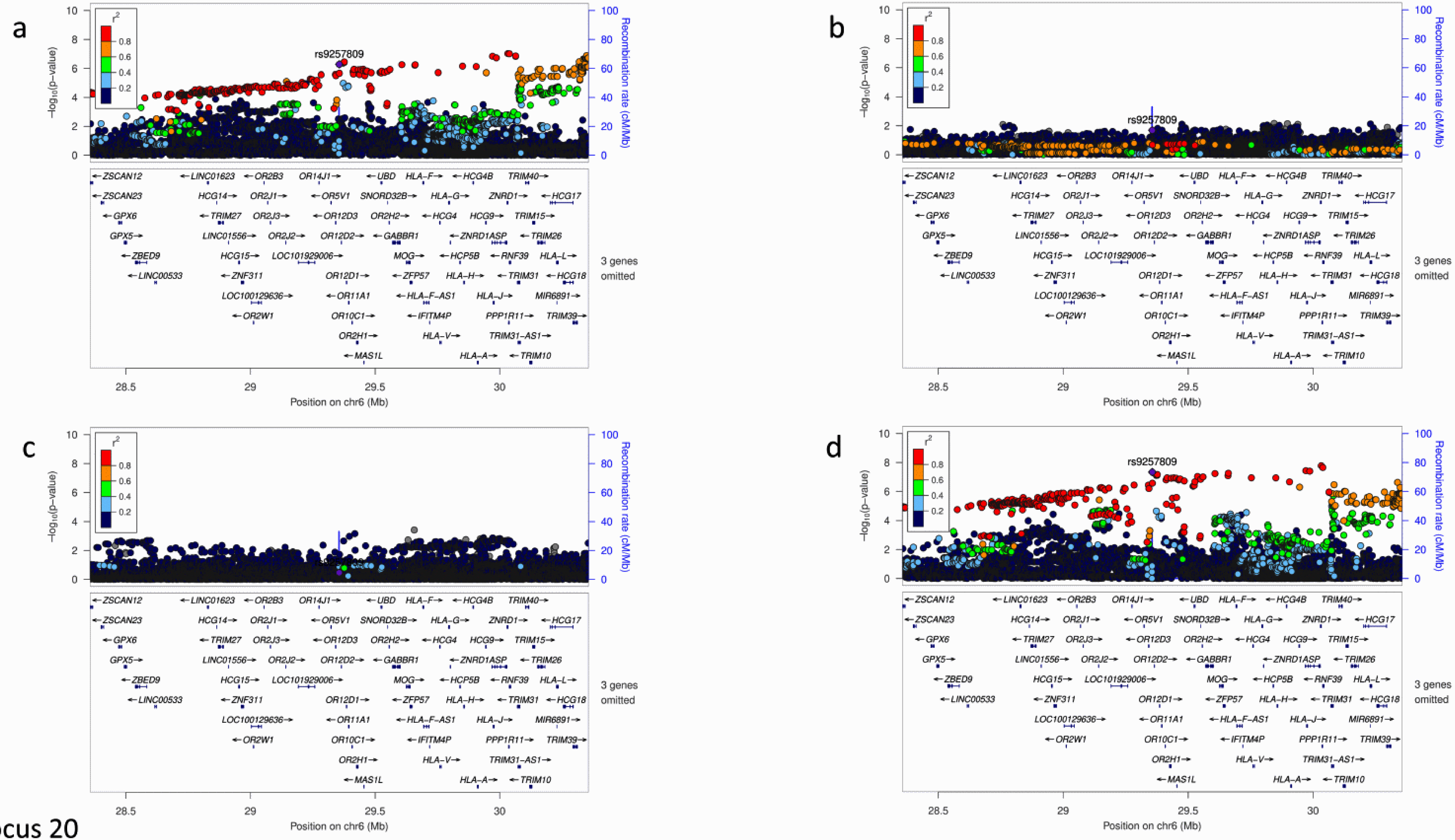
a
b
c
d
r2
0.8
0.6
0.4
0.2
rs9257809
−log10(p−value)
Recombination rate (cM/Mb)
Position on chr6 (Mb)
28.5
29
29.5
30
3 genes omitted
ZSCAN12
ZSCAN23
GPX6
GPX5
ZBED9
LINC00533
LINC01623
HCG14
TRIM27
LINC01556
HCG15
ZNF311
LOC100129636
OR2W1
OR2B3
OR2J1
OR2J3
OR2J2
LOC101929006
OR14J1
OR5V1
OR12D3
OR12D2
OR12D1
OR11A1
OR10C1
OR2H1
MAS1L
UBD
SNORD32B
OR2H2
GABBR1
MOG
ZFP57
HLA−F−AS1
IFITM4P
HLA−V
HLA−F
HLA−G
HCG4
ZNRD1ASP
HCP5B
HLA−H
HLA−J
PPP1R11
TRIM31−AS1
HLA−A
HCG4B
HCG9
RNF39
TRIM31
ZNRD1
TRIM40
TRIM15
TRIM26
HLA−L
HCG18
MIR6891
TRIM39
TRIM10
HCG17

Locus 20

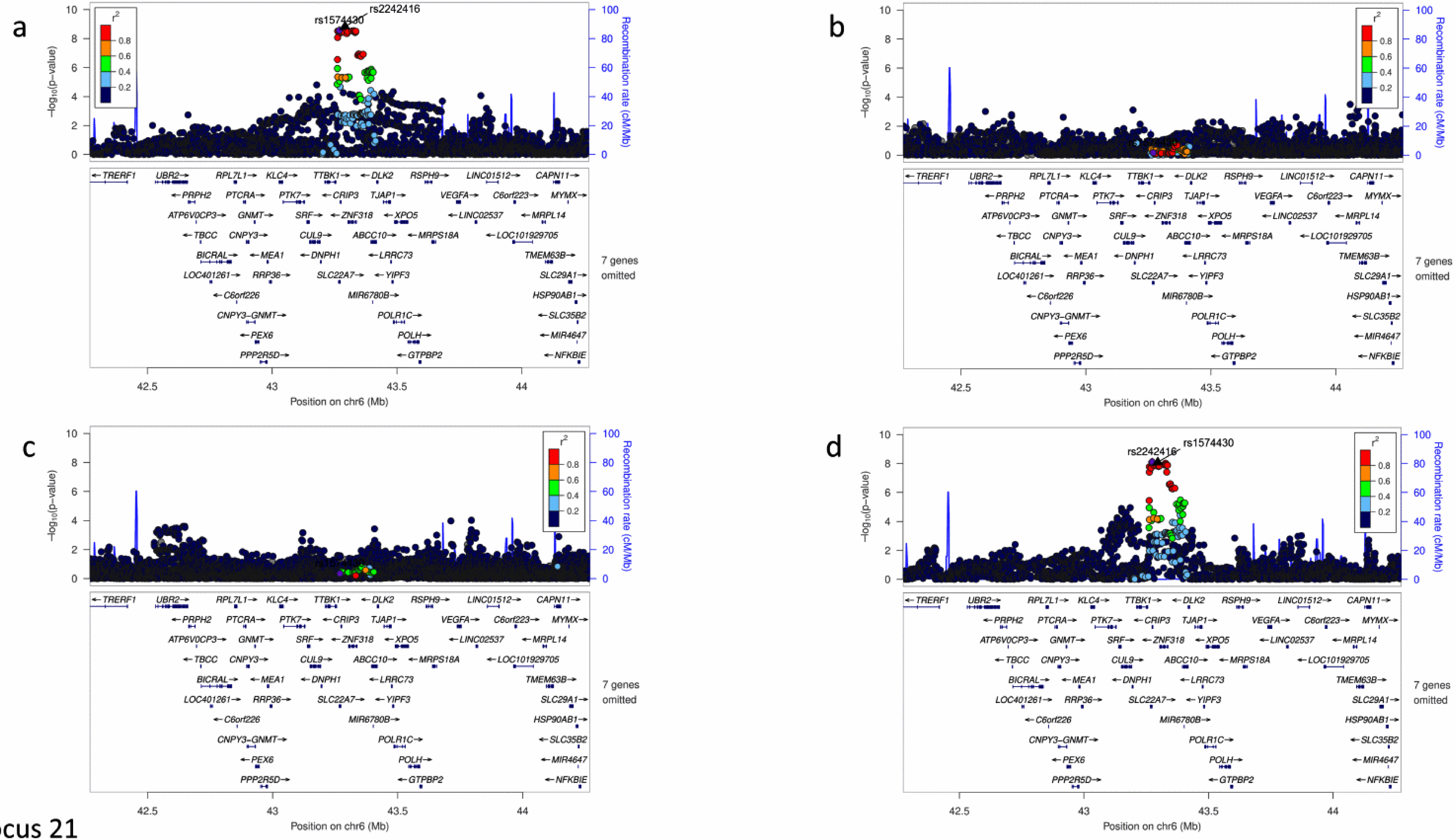
a
b
c
d
rs1574430
rs2242416
Position on chr6 (Mb)
Recombination rate (cM/Mb)
7 genes omitted

Locus 21

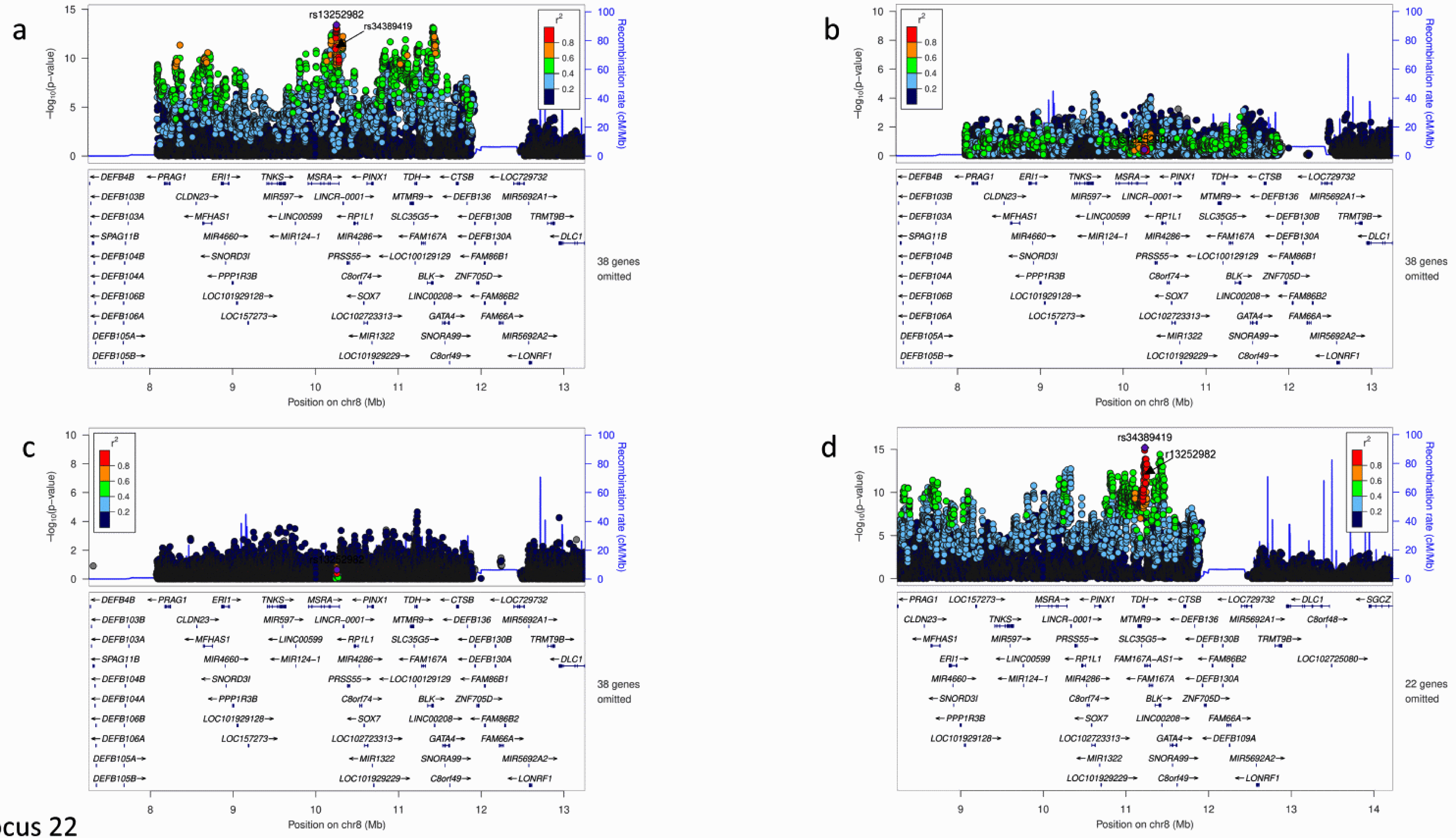
a
b
c
d
rs13252982
rs34389419
r13252982
r²
0.8
0.6
0.4
0.2
−log10(p−value)
Recombination rate (cM/Mb)
Position on chr8 (Mb)
38 genes omitted
22 genes omitted

Locus 22

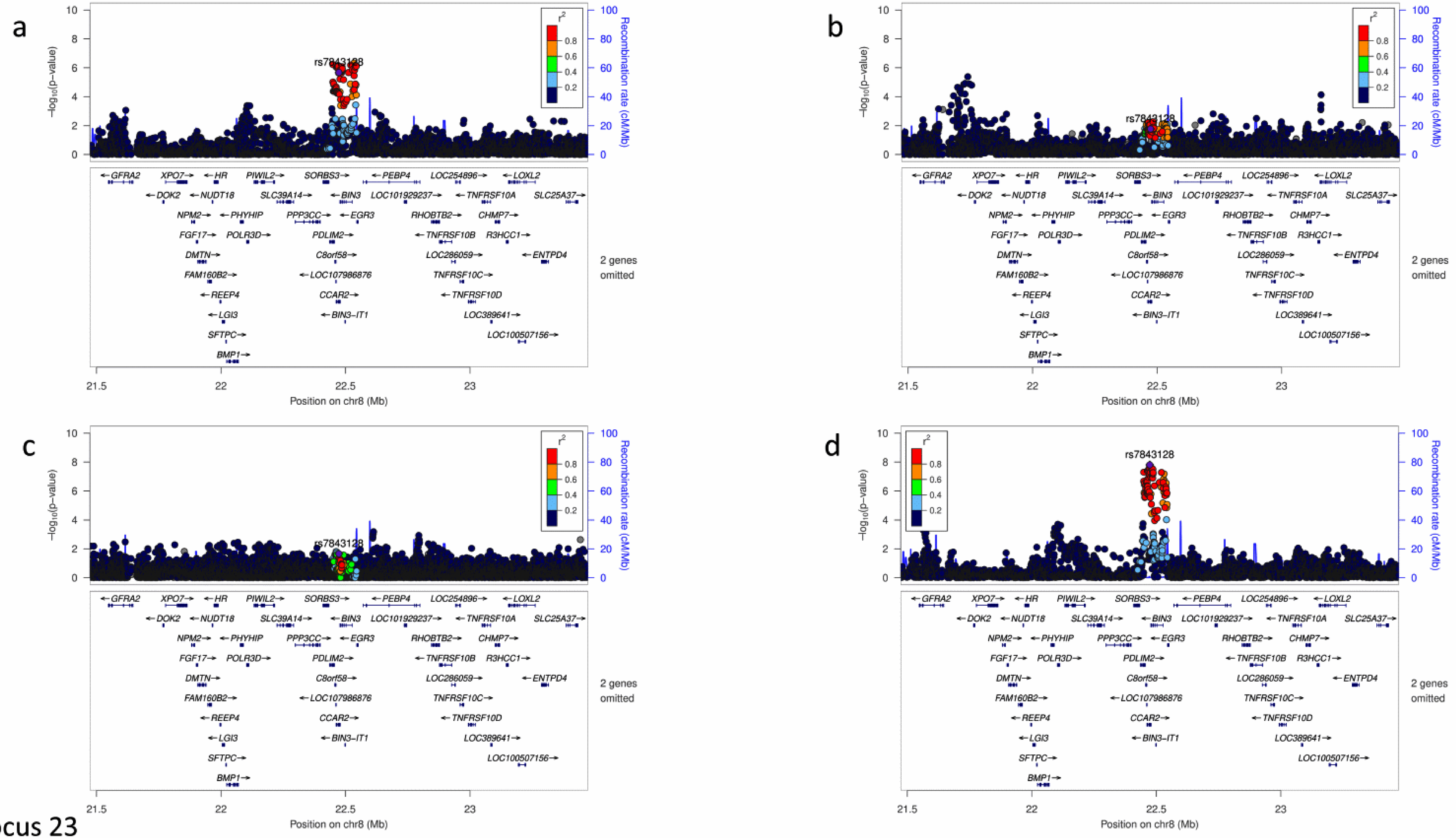
a
b
c
d
rs7843128
−log10(p−value)
Recombination rate (cM/Mb)
r2
Position on chr8 (Mb)
2 genes omitted
GFRA2
XPO7
HR
PIWIL2
SORBS3
PEBP4
LOC254896
LOXL2
DOK2
NUDT18
SLC39A14
BIN3
LOC101929237
TNFRSF10A
SLC25A37
NPM2
PHYHIP
PPP3CC
EGR3
RHOBTB2
CHMP7
FGF17
POLR3D
PDLIM2
TNFRSF10B
R3HCC1
DMTN
C8orf58
LOC286059
ENTPD4
FAM160B2
LOC107986876
TNFRSF10C
REEP4
CCAR2
TNFRSF10D
LGI3
BIN3−IT1
LOC389641
SFTPC
LOC100507156
BMP1

Locus 23

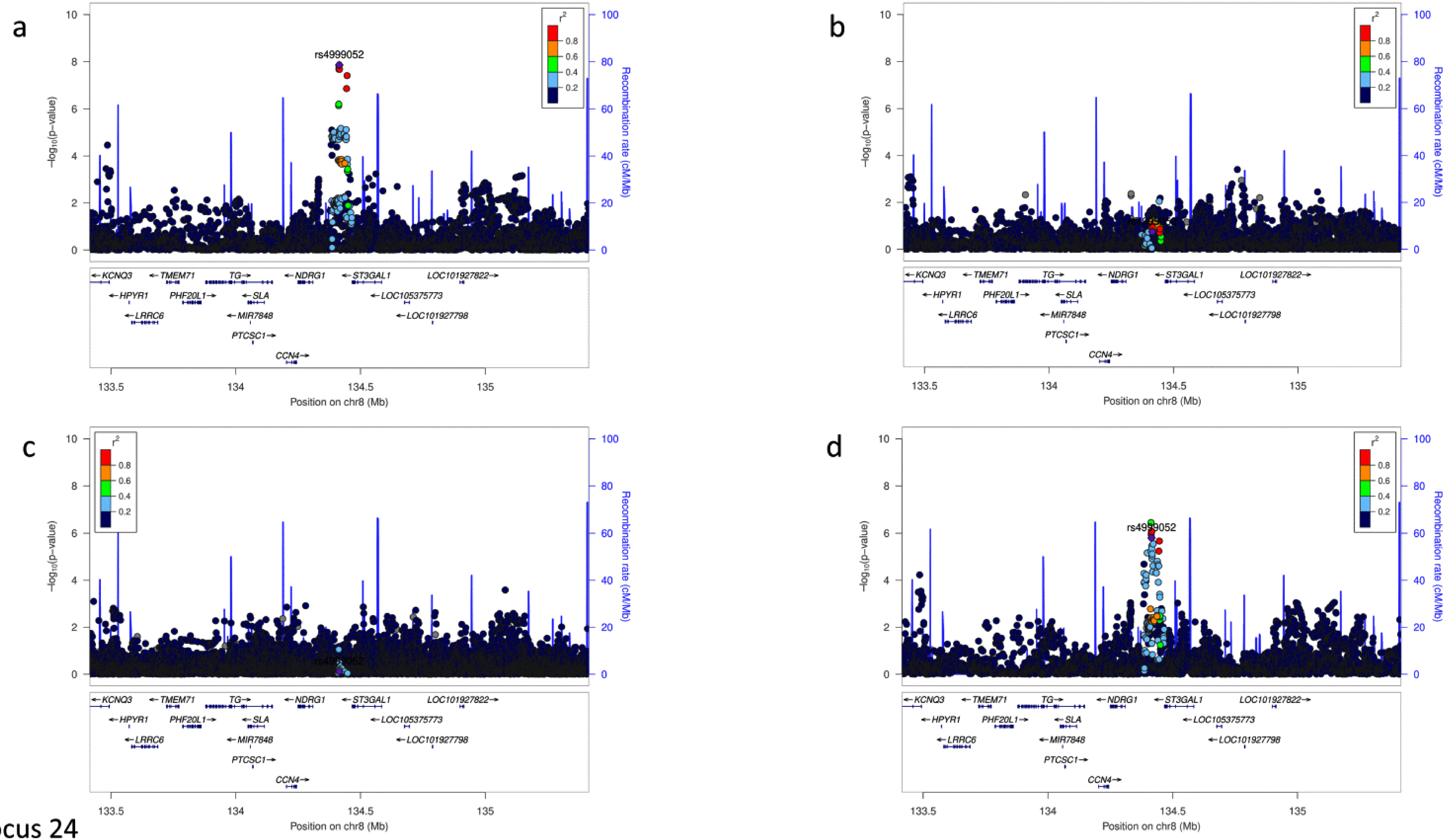
a
b
c
d
rs4999052
r2
0.8
0.6
0.4
0.2
−log10(p−value)
Recombination rate (cM/Mb)
KCNQ3
HPYR1
LRRC6
TMEM71
PHF20L1
TG
SLA
MIR7848
PTCSC1
CCN4
NDRG1
ST3GAL1
LOC105375773
LOC101927822
LOC101927798
Position on chr8 (Mb)
133.5
134
134.5
135

Locus 24

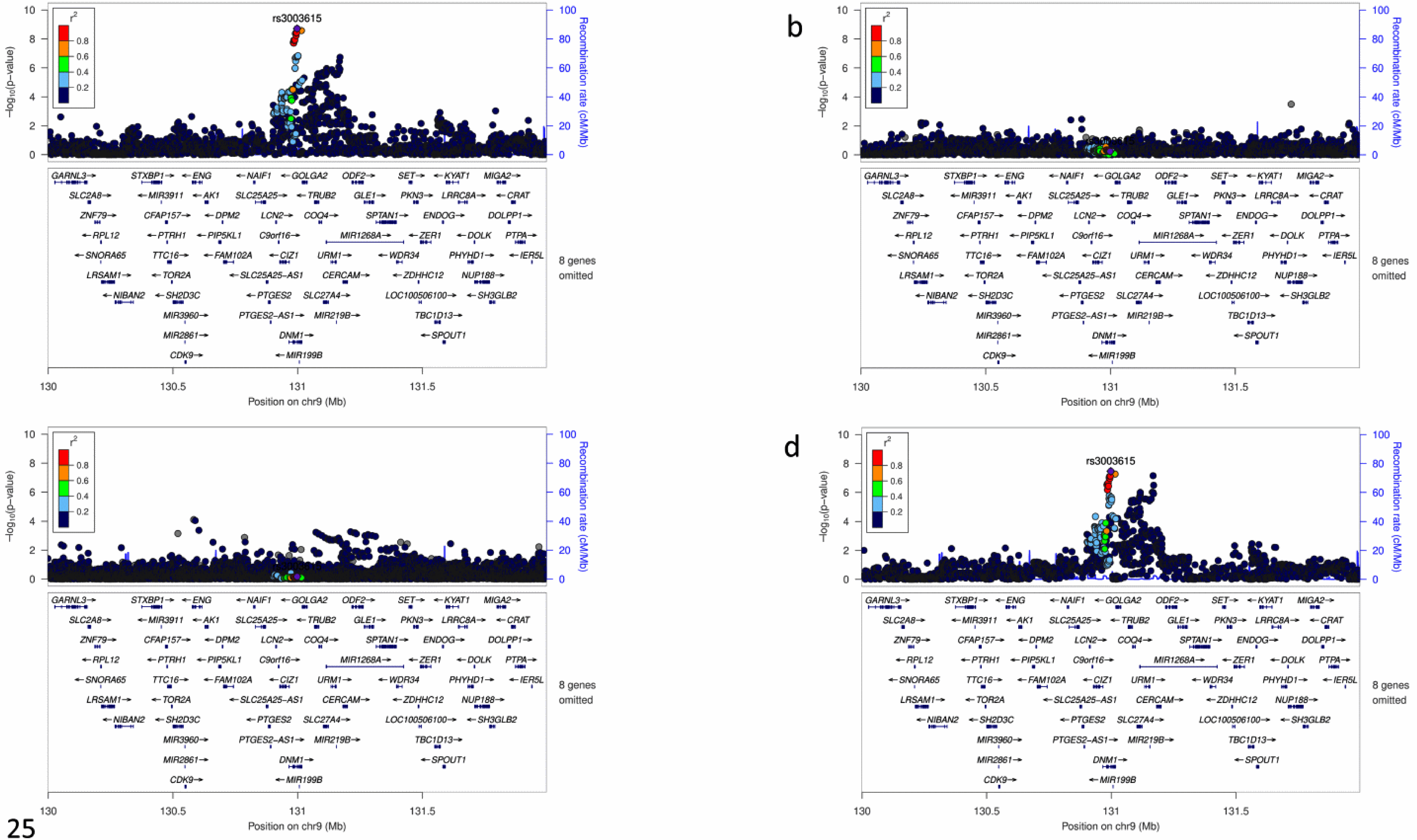
a
b
c
d
rs3003615
−log10(p−value)
Recombination rate (cM/Mb)
Position on chr9 (Mb)
r2
8 genes omitted

Locus 25

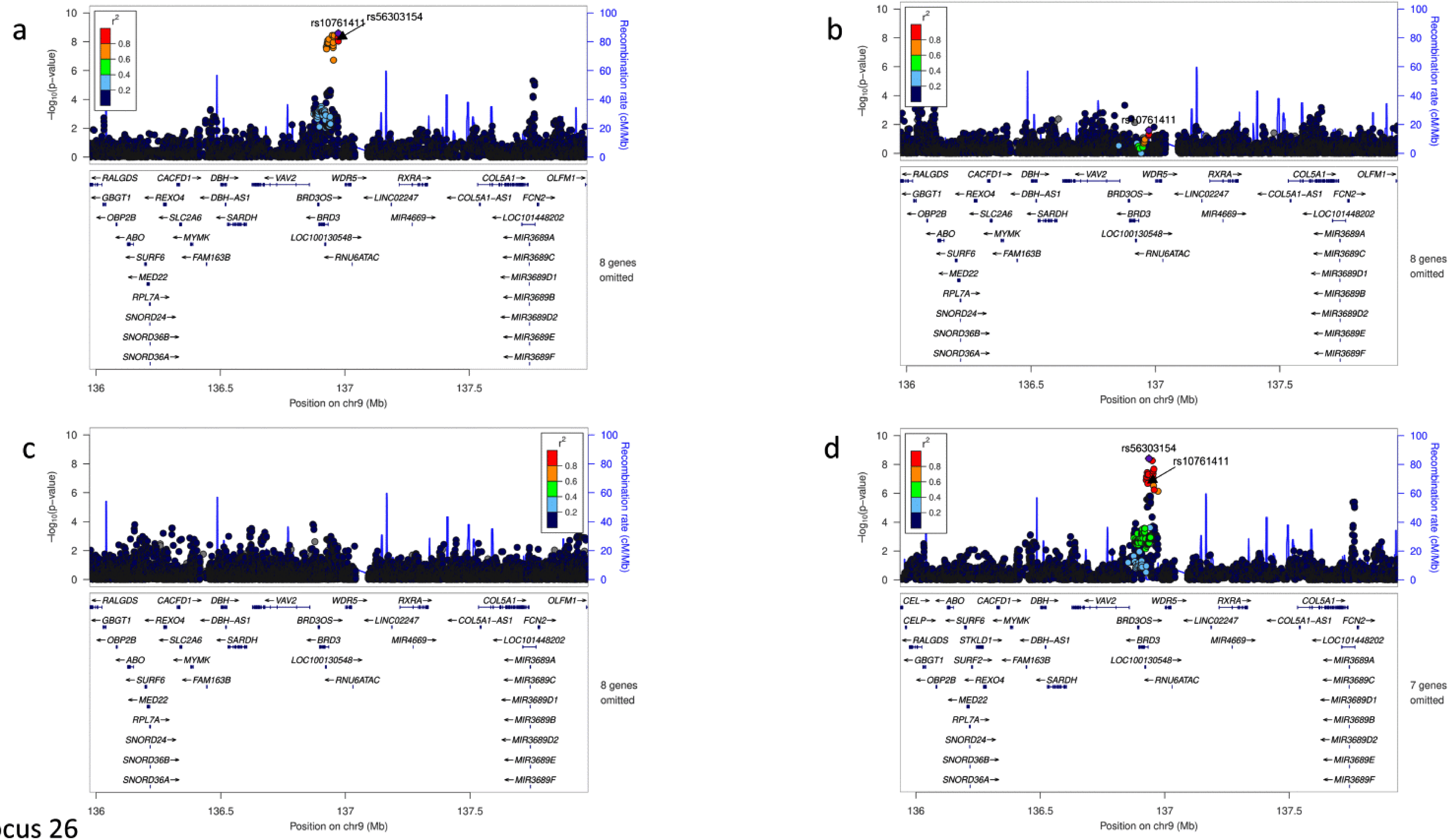
a
b
c
d
rs10761411
rs56303154
r2
0.8
0.6
0.4
0.2
−log10(p−value)
Recombination rate (cM/Mb)
Position on chr9 (Mb)
136
136.5
137
137.5
RALGDS
CACFD1
DBH
VAV2
WDR5
RXRA
COL5A1
OLFM1
GBGT1
REXO4
DBH−AS1
BRD3OS
LINC02247
COL5A1−AS1
FCN2
OBP2B
SLC2A6
SARDH
BRD3
MIR4669
LOC101448202
ABO
MYMK
LOC100130548
MIR3689A
SURF6
FAM163B
RNU6ATAC
MIR3689C
MED22
MIR3689D1
RPL7A
MIR3689B
SNORD24
MIR3689D2
SNORD36B
MIR3689E
SNORD36A
MIR3689F
8 genes omitted
CEL
CELP
STKLD1
SURF2
7 genes omitted

Locus 26

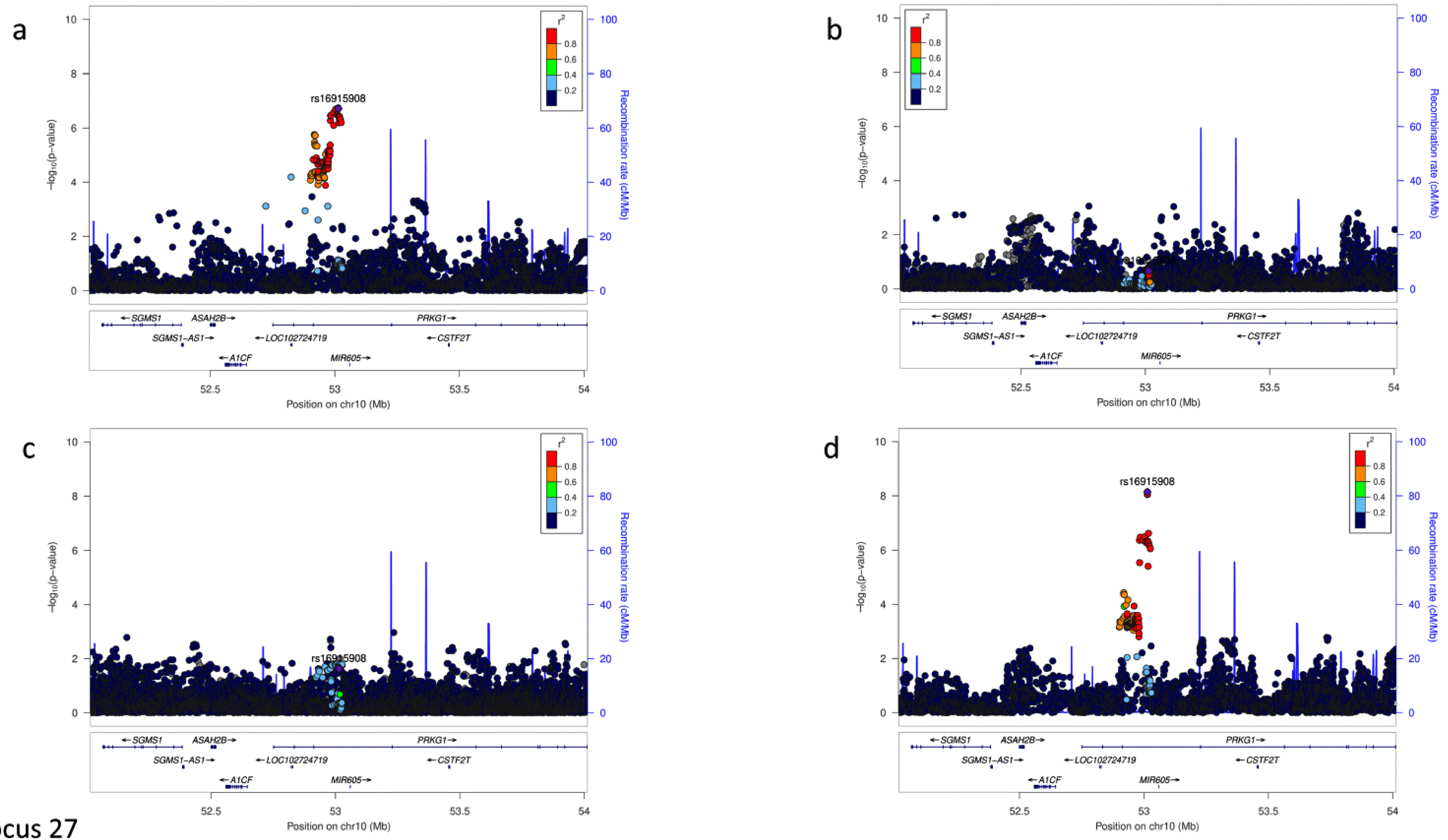
a
b
c
d
rs16915908
r²
0.8
0.6
0.4
0.2
-log10(p-value)
Recombination rate (cM/Mb)
Position on chr10 (Mb)
SGMS1
ASAH2B
PRKG1
SGMS1-AS1
LOC102724719
CSTF2T
A1CF
MIR605

Locus 27

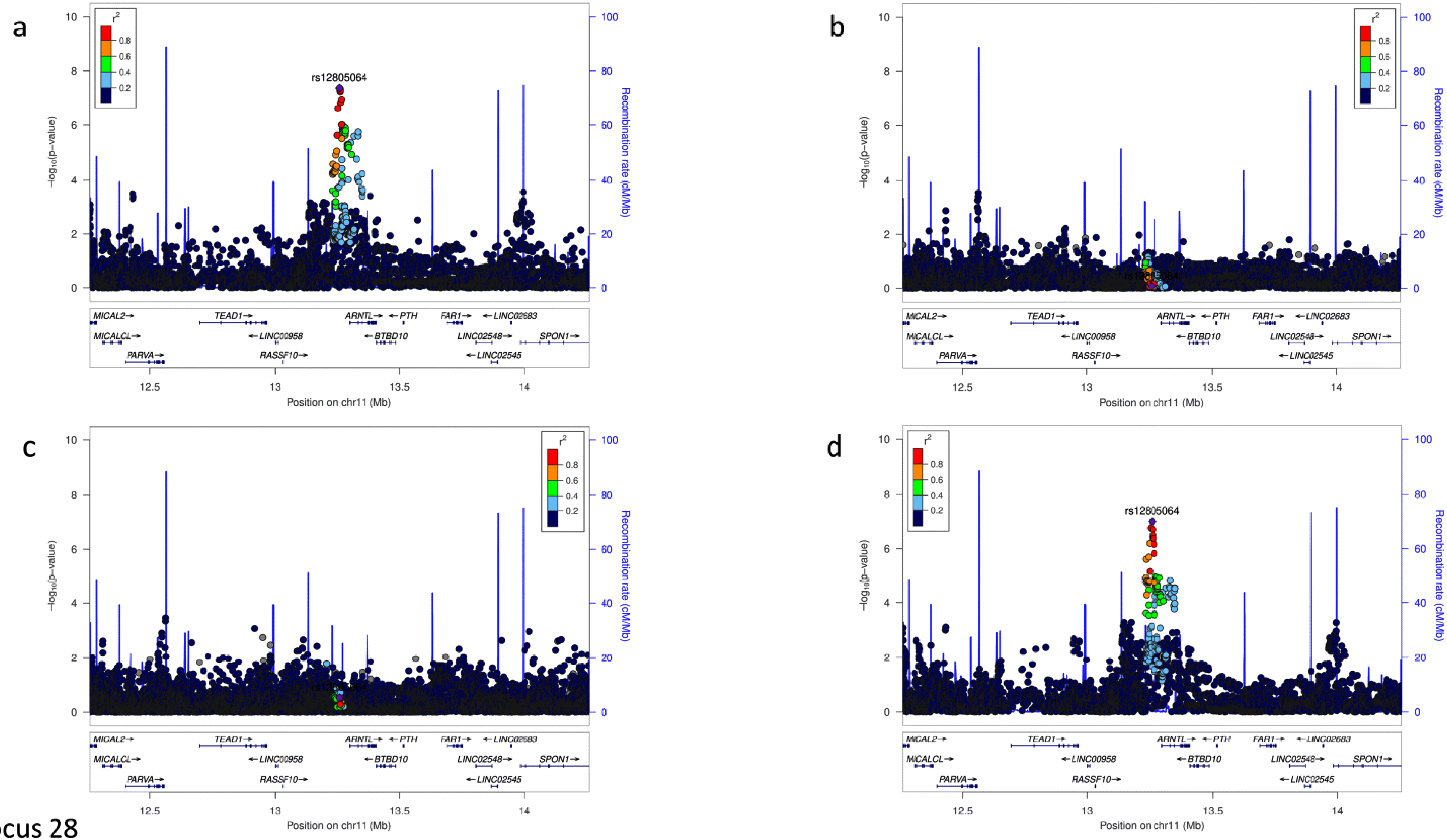
a
b
c
d
r²
0.8
0.6
0.4
0.2
rs12805064
−log10(p−value)
Recombination rate (cM/Mb)
Position on chr11 (Mb)
MICAL2→
MICALCL→
PARVA→
TEAD1→
←LINC00958
RASSF10→
ARNTL→
←PTH
←BTBD10
FAR1→
←LINC02683
LINC02548→
←LINC02545
SPON1→

Locus 28

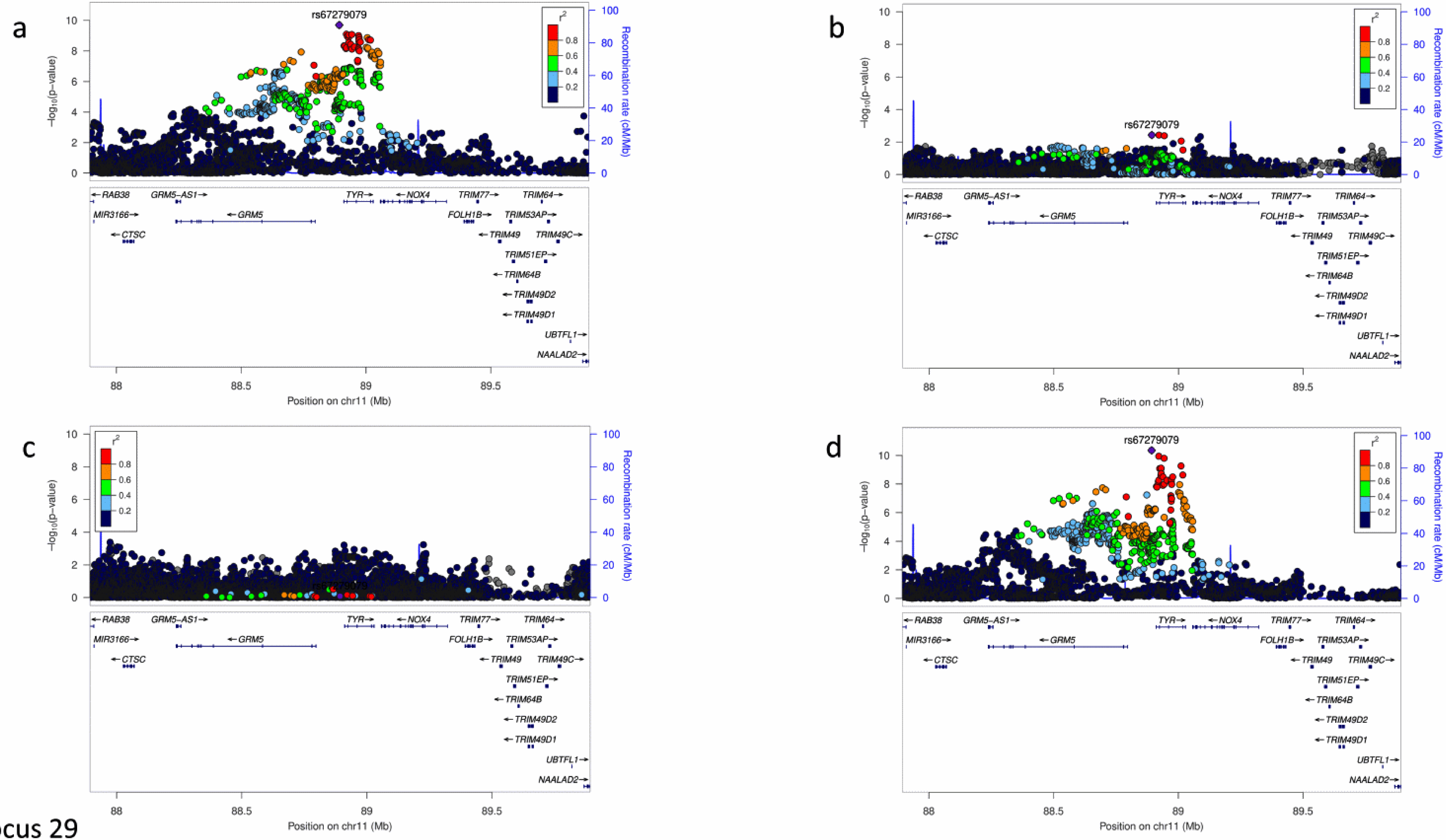
a
b
c
d
rs67279079
−log10(p-value)
Recombination rate (cM/Mb)
r2
0.8
0.6
0.4
0.2
Position on chr11 (Mb)
88
88.5
89
89.5
RAB38
MIR3166
CTSC
GRM5-AS1
GRM5
TYR
NOX4
TRIM77
FOLH1B
TRIM49
TRIM64
TRIM53AP
TRIM49C
TRIM51EP
TRIM64B
TRIM49D2
TRIM49D1
UBTFL1
NAALAD2

Locus 29

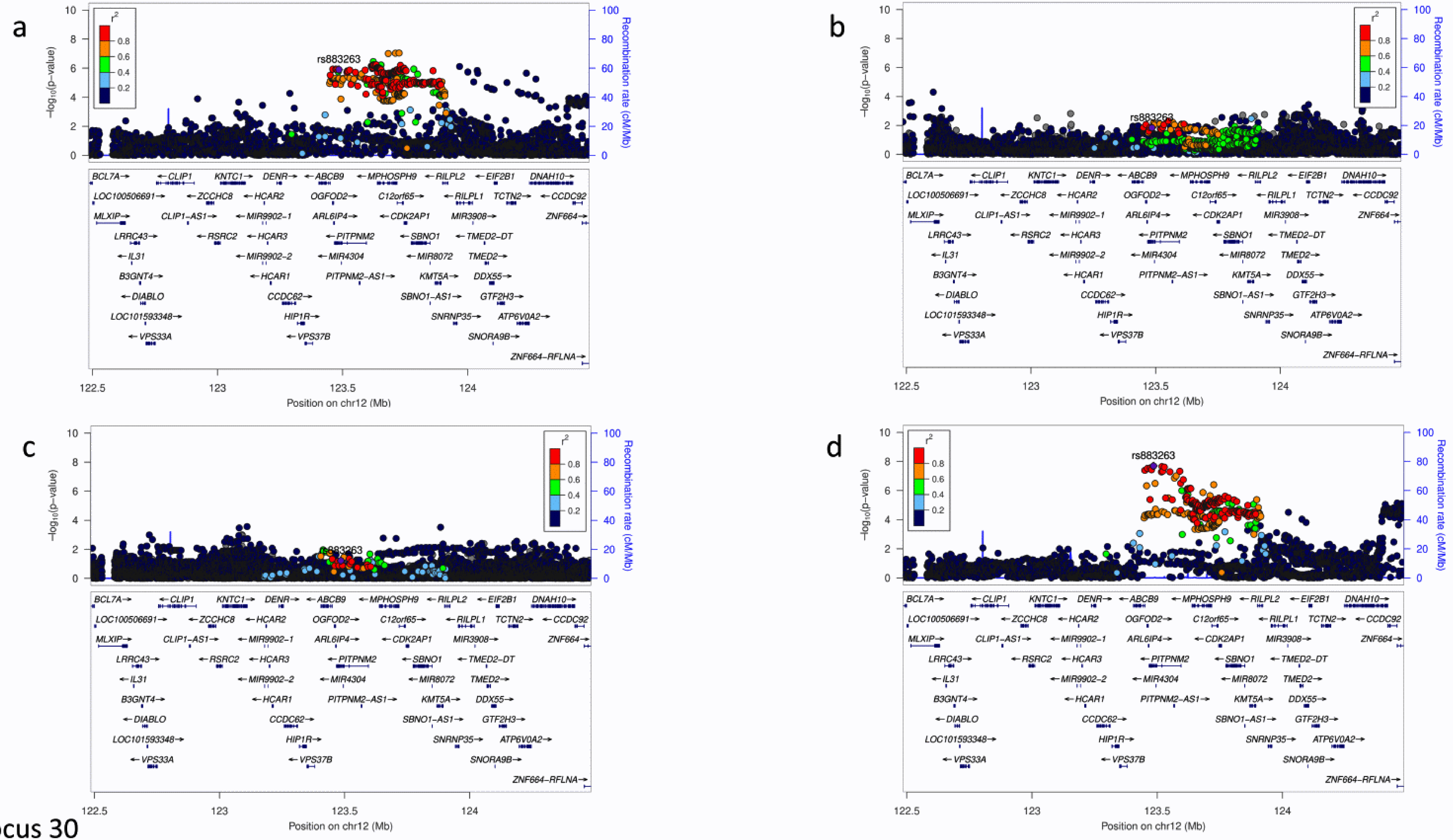
a
b
c
d
rs883263
−log10(p−value)
Recombination rate (cM/Mb)
Position on chr12 (Mb)
r2
BCL7A
CLIP1
KNTC1
DENR
ABCB9
MPHOSPH9
RILPL2
EIF2B1
DNAH10
LOC100506691
ZCCHC8
HCAR2
OGFOD2
C12orf65
RILPL1
TCTN2
CCDC92
MLXIP
CLIP1−AS1
MIR9902−1
ARL6IP4
CDK2AP1
MIR3908
ZNF664
LRRC43
RSRC2
HCAR3
PITPNM2
SBNO1
TMED2−DT
IL31
MIR9902−2
MIR4304
MIR8072
TMED2
B3GNT4
HCAR1
PITPNM2−AS1
KMT5A
DDX55
DIABLO
CCDC62
SBNO1−AS1
GTF2H3
LOC101593348
HIP1R
SNRNP35
ATP6V0A2
VPS33A
VPS37B
SNORA9B
ZNF664−RFLNA
Locus 30

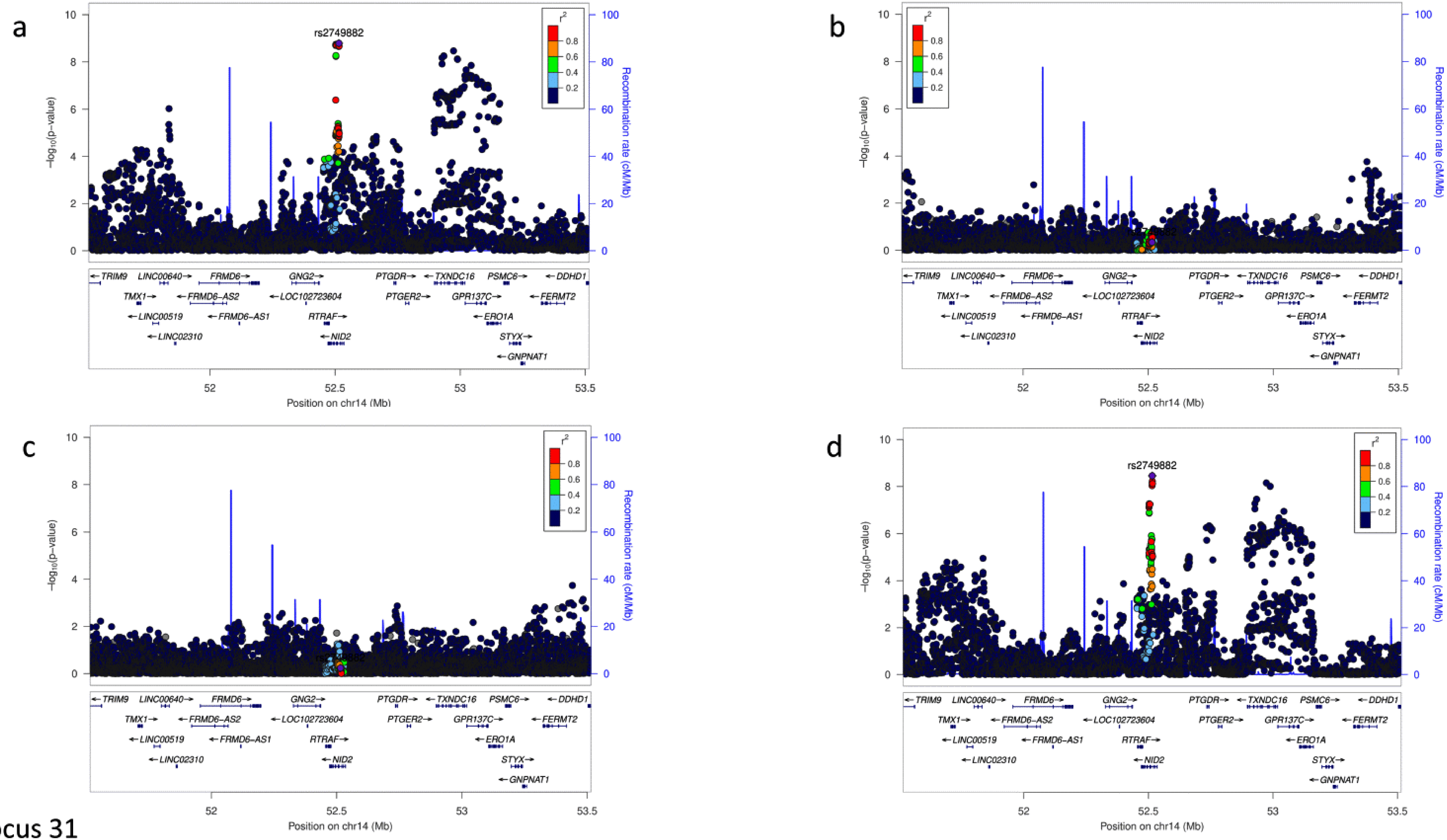
a
b
c
d
rs2749882
r2
0.8
0.6
0.4
0.2
−log10(p−value)
Recombination rate (cM/Mb)
Position on chr14 (Mb)
52
52.5
53
53.5
TRIM9
LINC00640
FRMD6
GNG2
PTGDR
TXNDC16
PSMC6
DDHD1
TMX1
FRMD6−AS2
LOC102723604
PTGER2
GPR137C
FERMT2
LINC00519
FRMD6−AS1
RTRAF
ERO1A
LINC02310
NID2
STYX
GNPNAT1

Locus 31

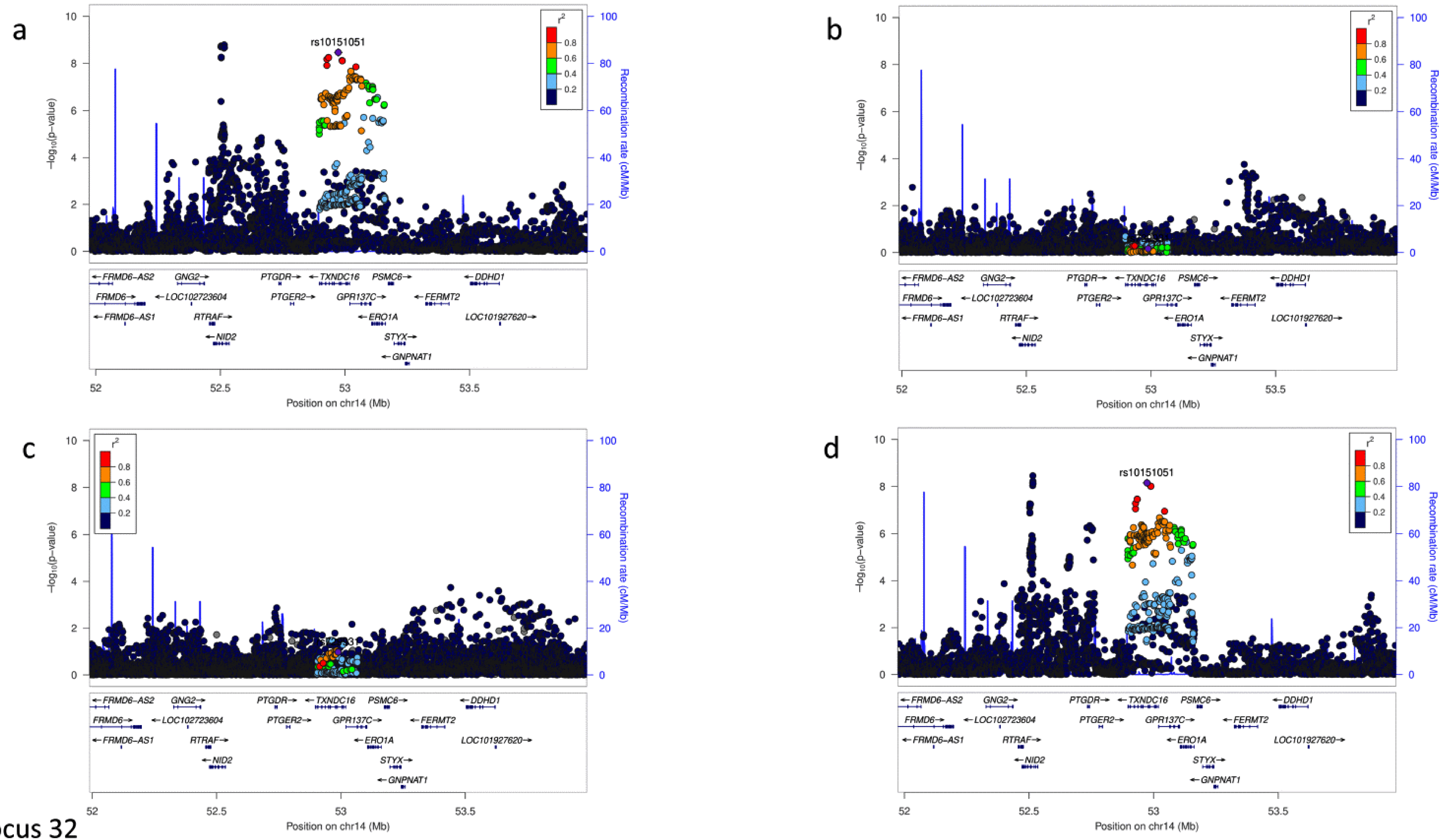
a
b
c
d
rs10151051
rs10151051
r2
-log10(p-value)
Recombination rate (cM/Mb)
Position on chr14 (Mb)
FRMD6-AS2
FRMD6
FRMD6-AS1
GNG2
LOC102723604
RTRAF
NID2
PTGDR
PTGER2
TXNDC16
GPR137C
ERO1A
STYX
GNPNAT1
PSMC6
FERMT2
DDHD1
LOC101927620

Locus 32

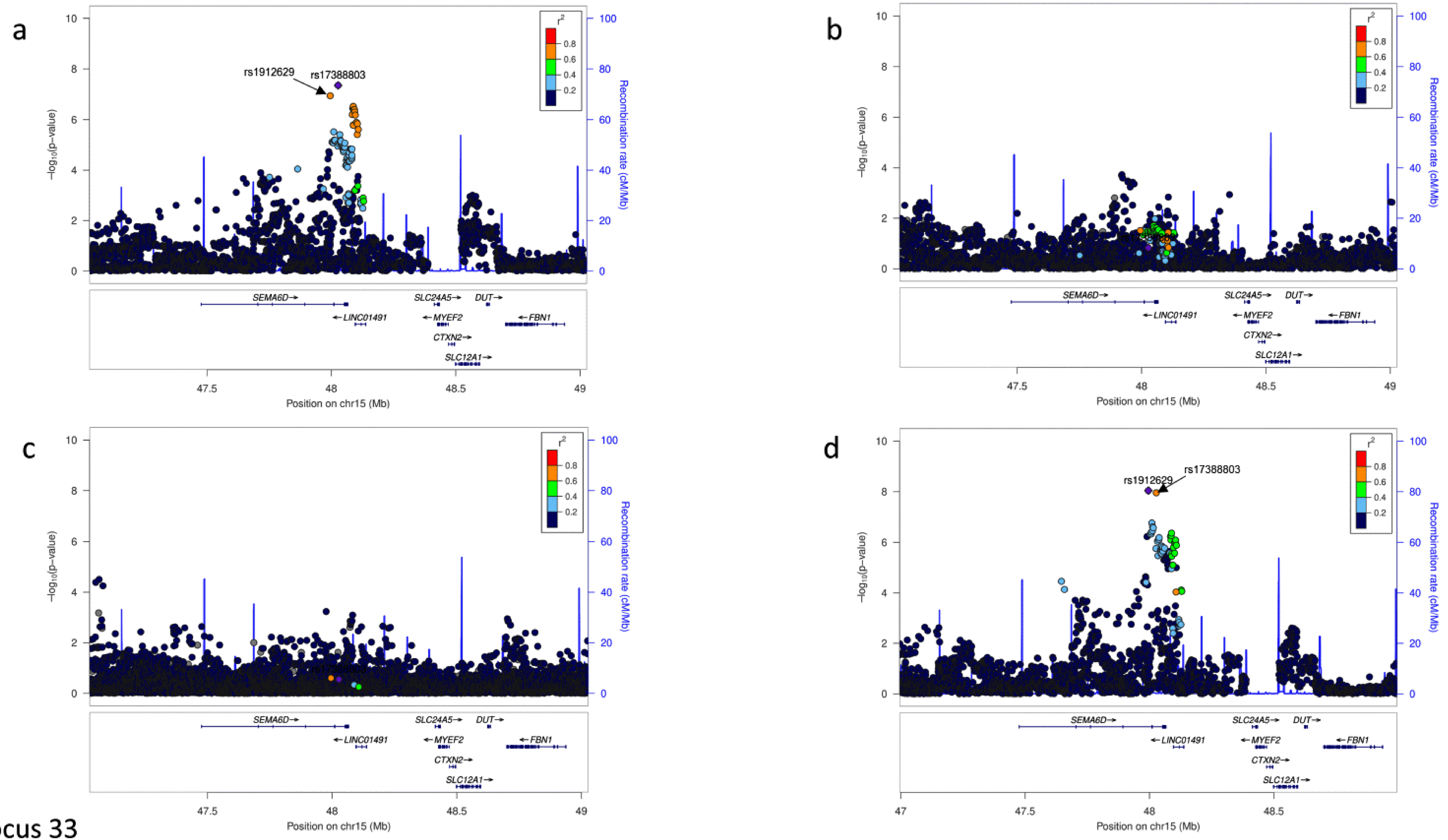
a
b
c
d
rs1912629
rs17388803
−log10(p−value)
Recombination rate (cM/Mb)
r2
SEMA6D
LINC01491
SLC24A5
MYEF2
CTXN2
SLC12A1
DUT
FBN1
Position on chr15 (Mb)

Locus 33

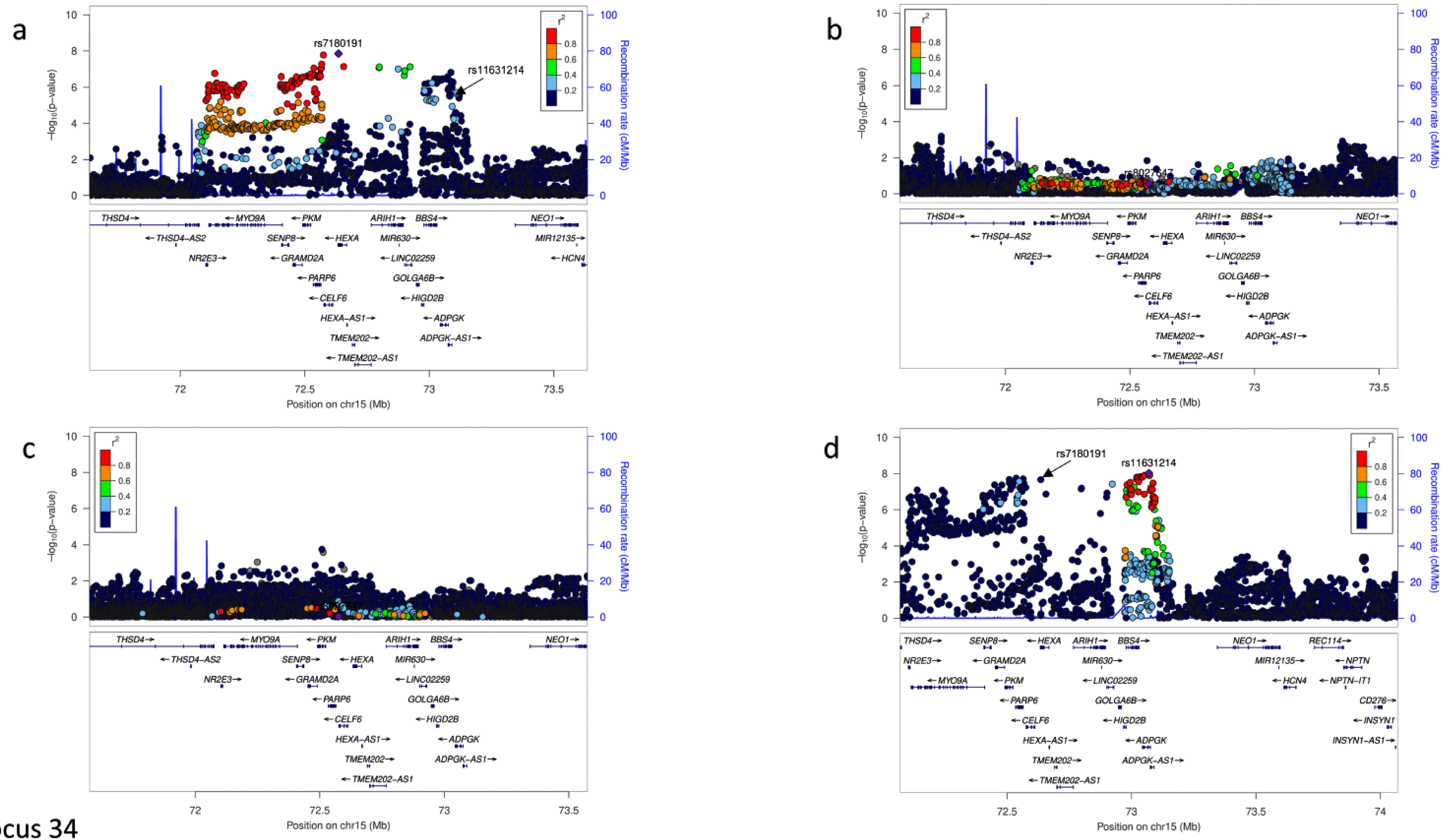
a
rs7180191
rs11631214
−log10(p−value)
Recombination rate (cM/Mb)
r²
THSD4→
←THSD4−AS2
NR2E3→
←MYO9A
SENP8→
←GRAMD2A
←PKM
←PARP6
←CELF6
←HEXA
HEXA−AS1→
TMEM202→
←TMEM202−AS1
ARIH1→
MIR630→
←LINC02259
GOLGA6B→
←HIGD2B
←ADPGK
ADPGK−AS1→
BBS4→
NEO1→
MIR12135→
←HCN4
Position on chr15 (Mb)
b
rs8027647
c
d
REC114→
←NPTN
←NPTN−IT1
CD276→
←INSYN1
INSYN1−AS1→

Locus 34

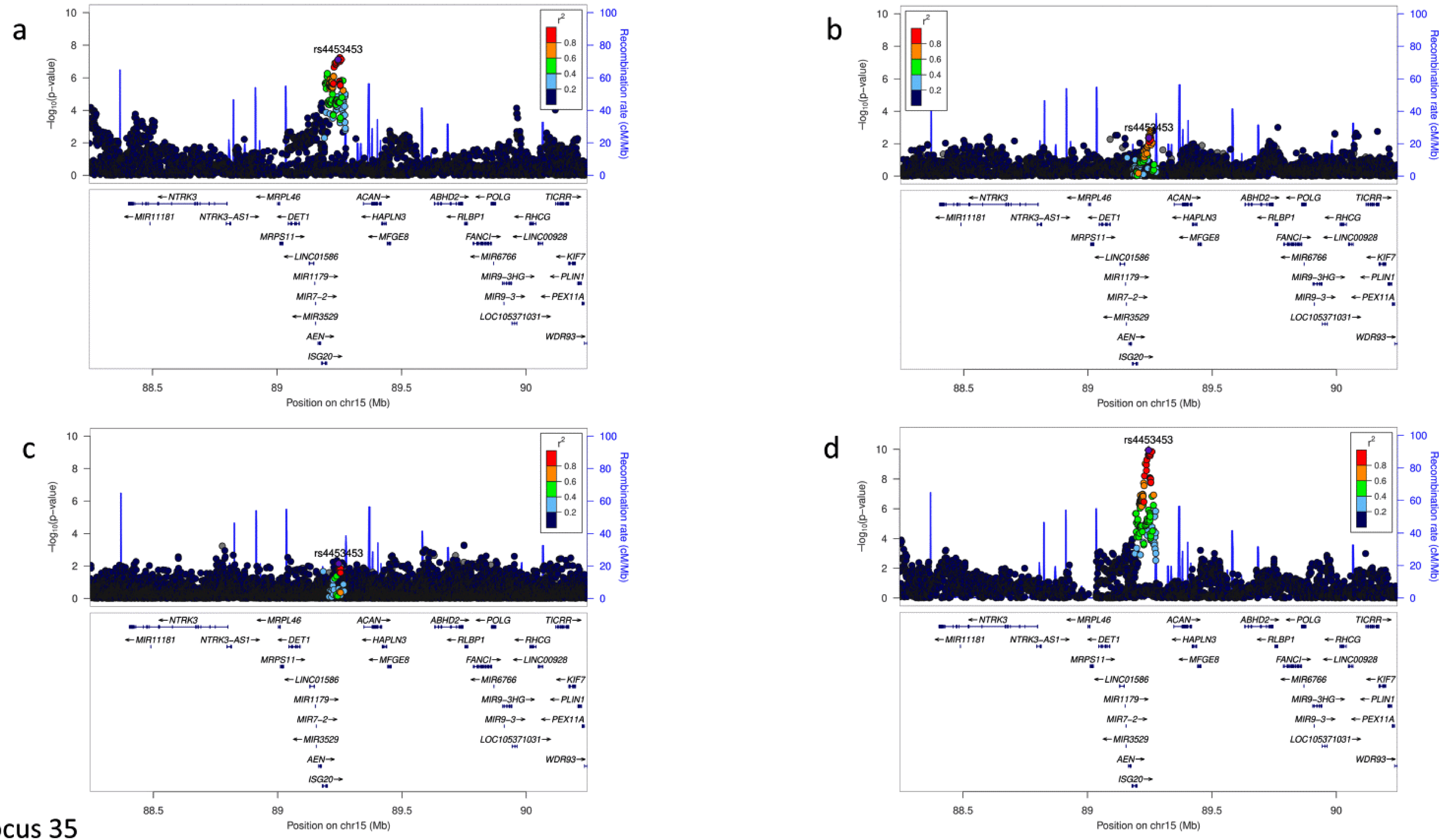
a
b
c
d
rs4453453
−log10(p−value)
Recombination rate (cM/Mb)
r2
0.8
0.6
0.4
0.2
Position on chr15 (Mb)
88.5
89
89.5
90
NTRK3
MRPL46
ACAN
ABHD2
POLG
TICRR
MIR11181
NTRK3−AS1
DET1
HAPLN3
RLBP1
RHCG
MRPS11
MFGE8
FANCI
LINC00928
LINC01586
MIR6766
KIF7
MIR1179
MIR9−3HG
PLIN1
MIR7−2
MIR9−3
PEX11A
MIR3529
LOC105371031
AEN
WDR93
ISG20

Locus 35

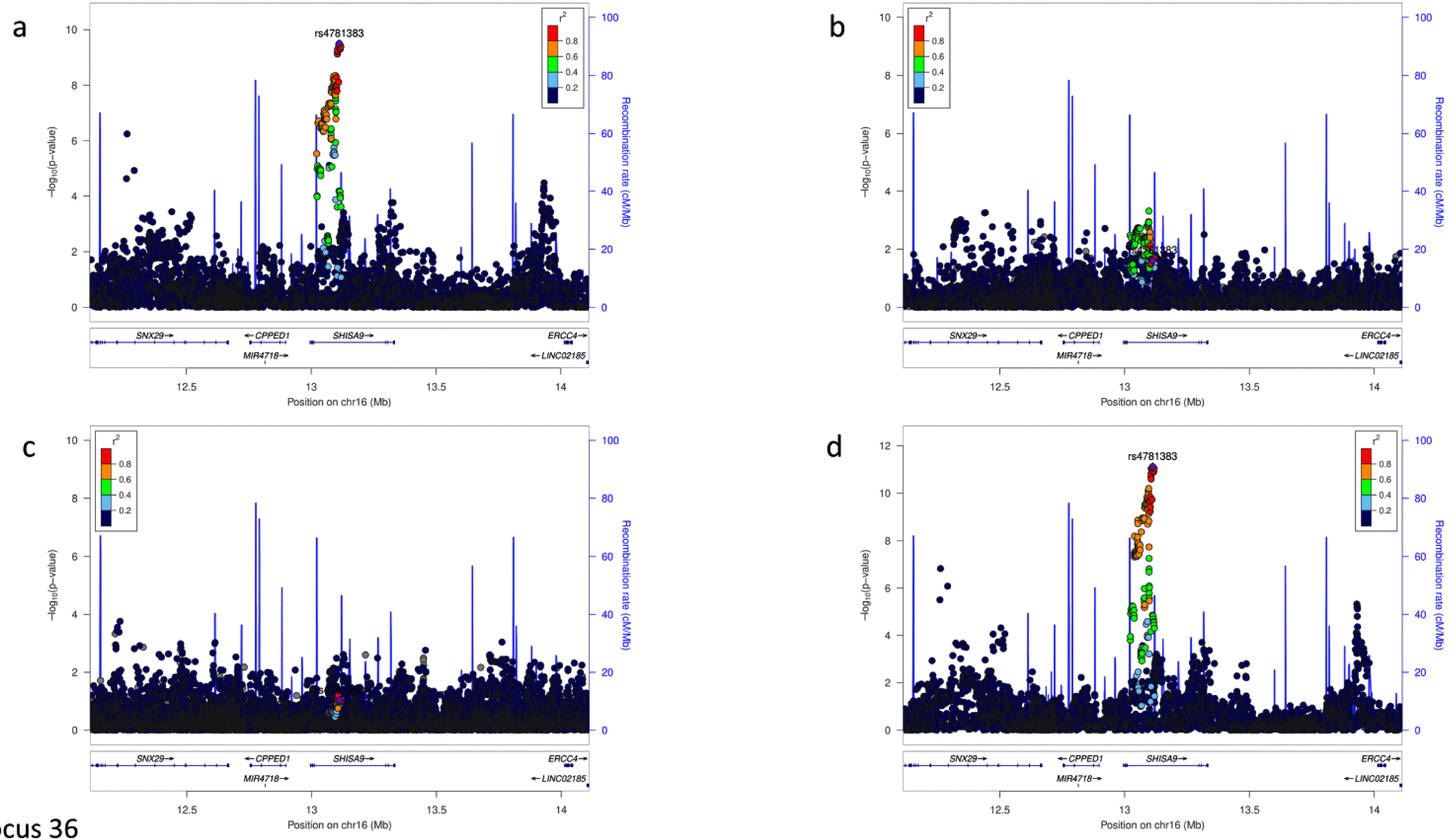
a
b
c
d
rs4781383
r2
0.8
0.6
0.4
0.2
−log10(p−value)
Recombination rate (cM/Mb)
SNX29→
←CPPED1
SHISA9→
ERCC4→
MIR4718→
←LINC02185
12.5
13
13.5
14
Position on chr16 (Mb)

Locus 36

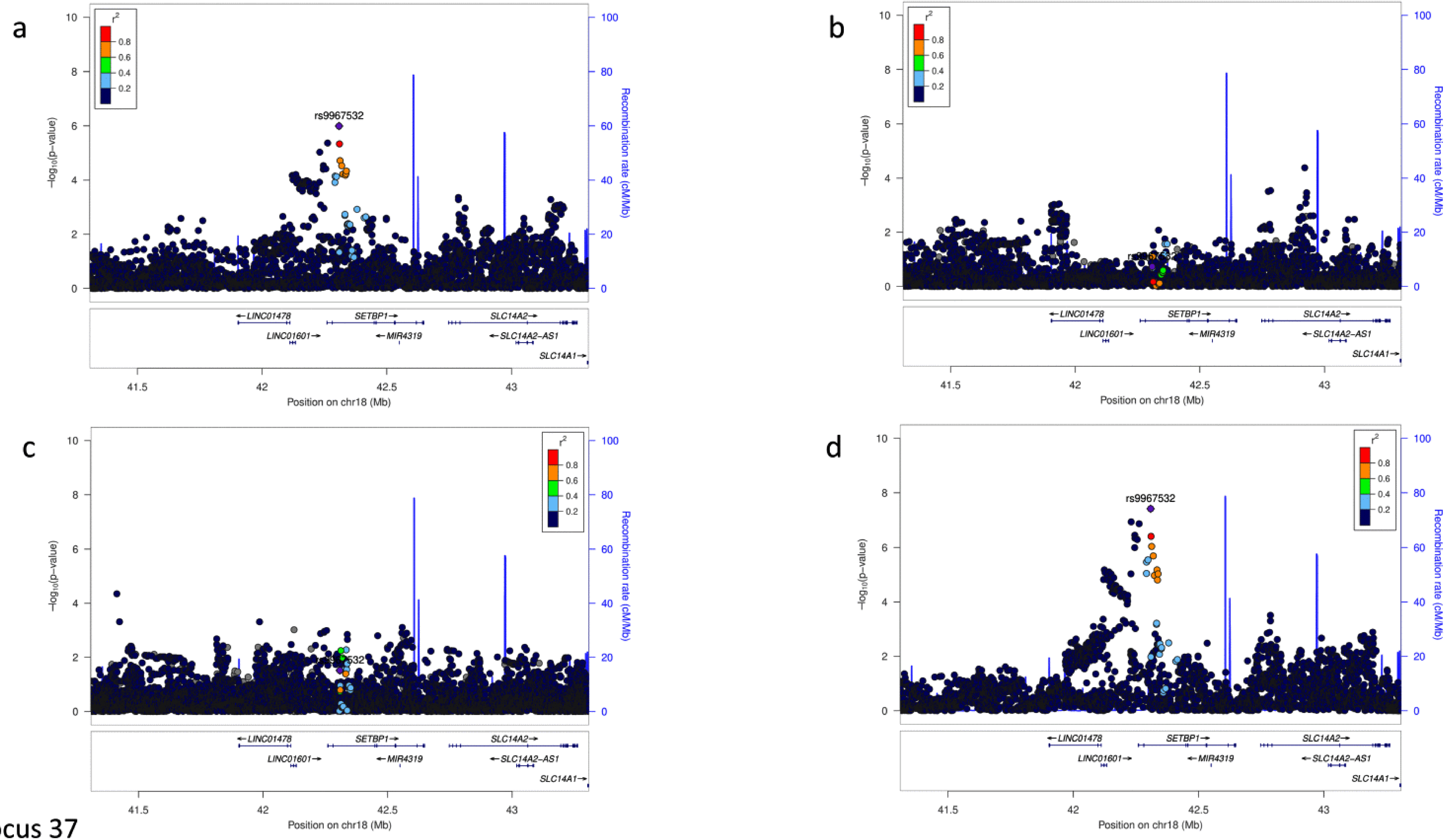
a
b
c
d
rs9967532
−log10(p−value)
Recombination rate (cM/Mb)
Position on chr18 (Mb)
r2
0.8
0.6
0.4
0.2
LINC01478
SETBP1
SLC14A2
LINC01601
MIR4319
SLC14A2−AS1
SLC14A1

Locus 37

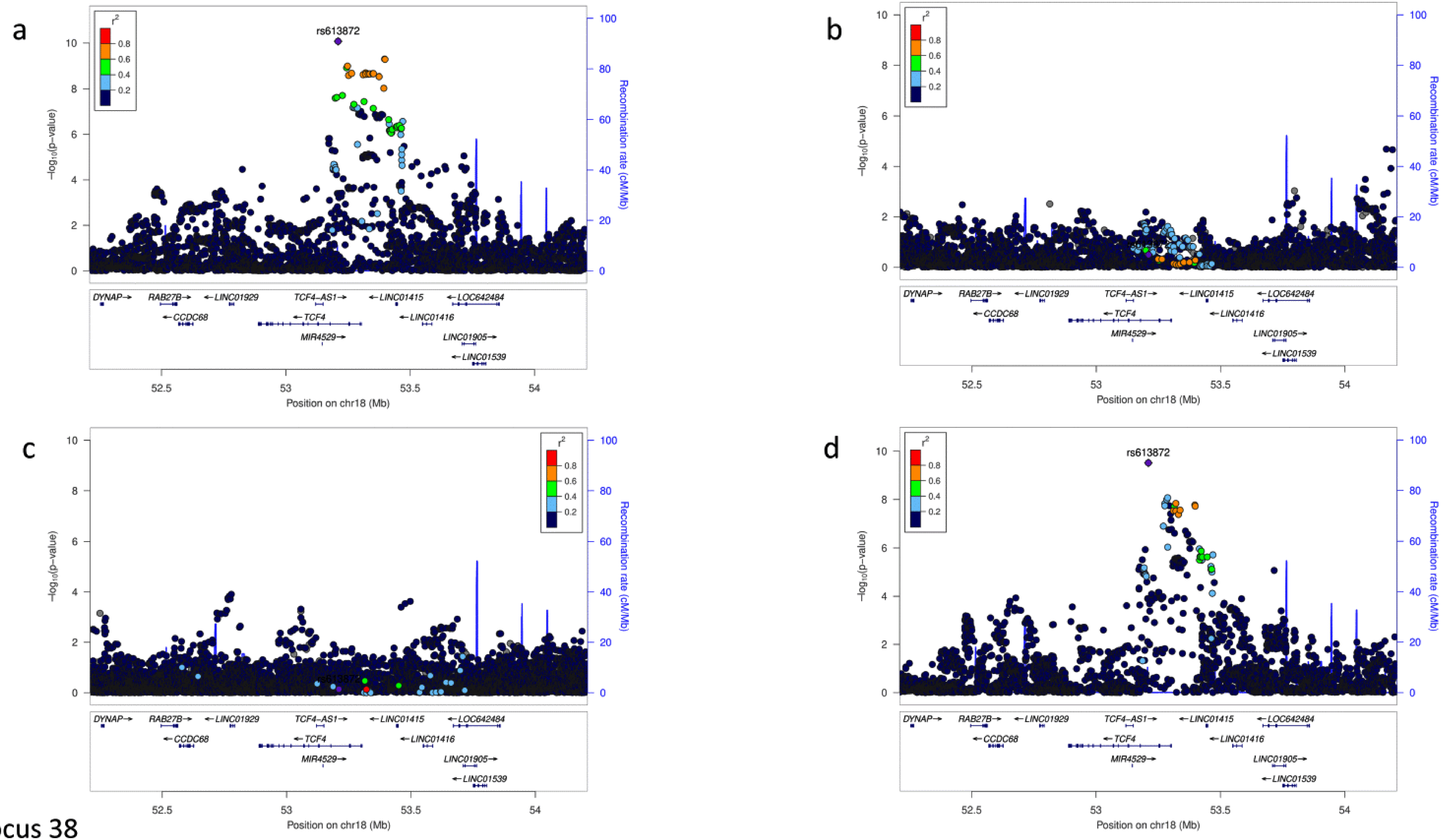
a
b
c
d
rs613872
r2
0.8
0.6
0.4
0.2
−log10(p−value)
Recombination rate (cM/Mb)
Position on chr18 (Mb)
DYNAP→
RAB27B→
←LINC01929
TCF4−AS1→
←LINC01415
←LOC642484
←CCDC68
←TCF4
←LINC01416
MIR4529→
LINC01905→
←LINC01539
52.5
53
53.5
54

Locus 38

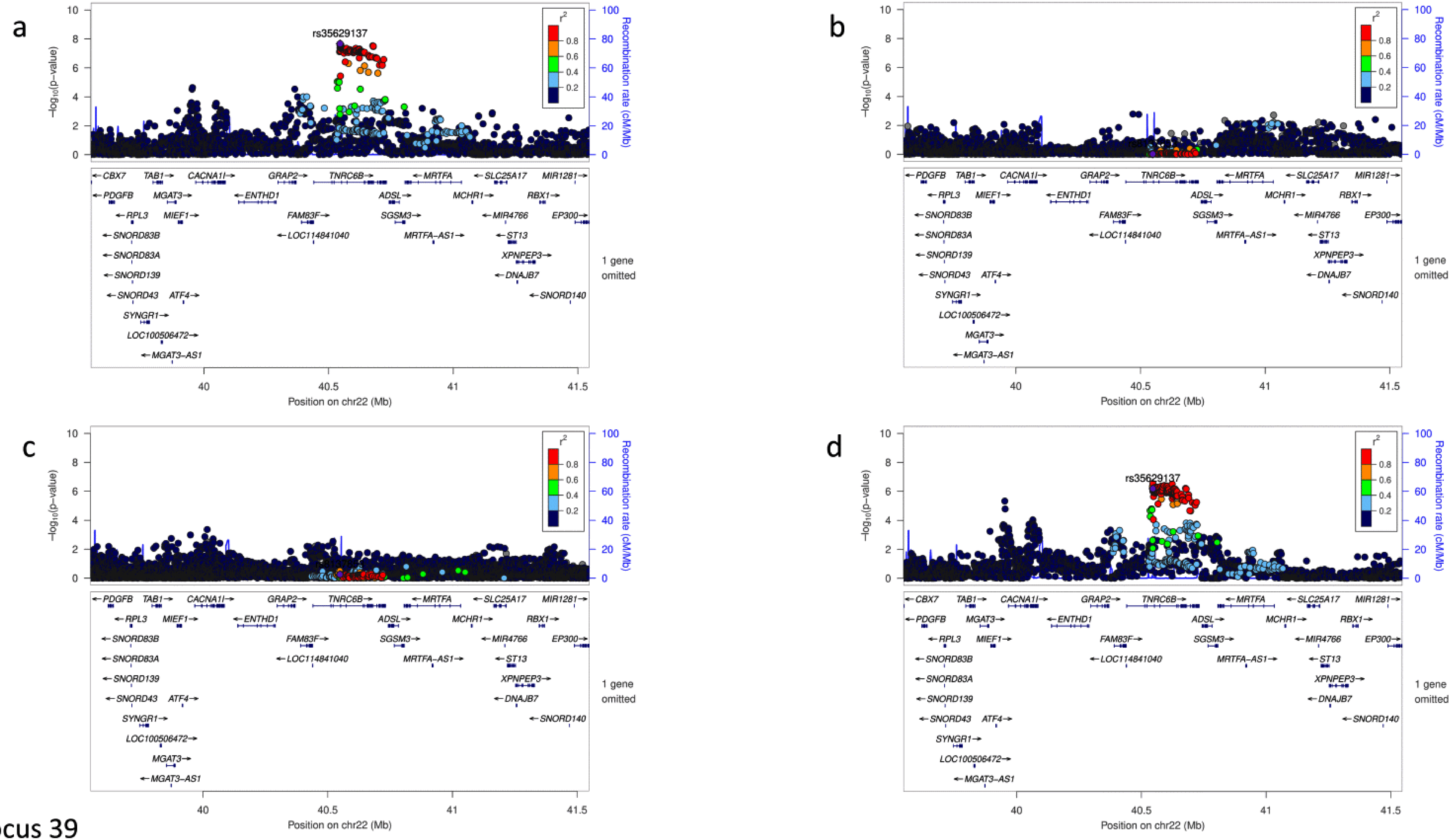

a
b
c
d
rs35629137
rs35629137
−log10(p−value)
Recombination rate (cM/Mb)
r2
0.8
0.6
0.4
0.2
Position on chr22 (Mb)
40
40.5
41
41.5
CBX7
PDGFB
RPL3
SNORD83B
SNORD83A
SNORD139
SNORD43
SYNGR1
LOC100506472
MGAT3-AS1
TAB1
MGAT3
MIEF1
ATF4
CACNA1I
ENTHD1
GRAP2
FAM83F
LOC114841040
TNRC6B
ADSL
SGSM3
MRTFA
MRTFA-AS1
MCHR1
SLC25A17
MIR4766
ST13
XPNPEP3
DNAJB7
MIR1281
RBX1
EP300
SNORD140
1 gene omitted

Locus 39
