# Supplementary material for: Genetic architecture distinguishes tinnitus from hearing loss
Source: Nat Commun. 2024 Jan 19;15:614. doi: 10.1038/s41467-024-44842-x (PMC10799010; doi:10.1038/s41467-024-44842-x)
Supplement: Supplementary file 25 — Reporting Summary [file 41467_2024_44842_MOESM25_ESM.pdf]

nature portfolio

Corresponding author(s): Caroline Nievergelt, Royce Clifford

Last updated by author(s): Nov 14, 2023

Reporting Summary

Nature Portfolio wishes to improve the reproducibility of the work that we publish. This form provides structure for consistency and transparency in reporting. For further information on Nature Portfolio policies, see our Editorial Policies and the Editorial Policy Checklist.

Statistics

For all statistical analyses, confirm that the following items are present in the figure legend, table legend, main text, or Methods section.

n/a	Confirmed	
☐	☒	The exact sample size (*n*) for each experimental group/condition, given as a discrete number and unit of measurement
☐	☒	A statement on whether measurements were taken from distinct samples or whether the same sample was measured repeatedly
☐	☒	The statistical test(s) used AND whether they are one- or two-sided<br>*Only common tests should be described solely by name; describe more complex techniques in the Methods section.*
☐	☒	A description of all covariates tested
☐	☒	A description of any assumptions or corrections, such as tests of normality and adjustment for multiple comparisons
☐	☒	A full description of the statistical parameters including central tendency (e.g. means) or other basic estimates (e.g. regression coefficient) AND variation (e.g. standard deviation) or associated estimates of uncertainty (e.g. confidence intervals)
☐	☒	For null hypothesis testing, the test statistic (e.g. *F*, *t*, *r*) with confidence intervals, effect sizes, degrees of freedom and *P* value noted<br>*Give P values as exact values whenever suitable.*
☒	☐	For Bayesian analysis, information on the choice of priors and Markov chain Monte Carlo settings
☒	☐	For hierarchical and complex designs, identification of the appropriate level for tests and full reporting of outcomes
☐	☒	Estimates of effect sizes (e.g. Cohen's *d*, Pearson's *r*), indicating how they were calculated

Our web collection on statistics for biologists contains articles on many of the points above.

Software and code

Policy information about availability of computer code

Data collection	No specific software was used for data collection in this study.
Data analysis	UKB phasing and imputation was done with SHAPEIT3 (reference panel: 1KGPp3) and IMPUTE (reference panel: combination of 1KGPp3, UK10K, Haplotype Reference Consortium), respectively. Ancestry was estimated using SNPweights with 2,027 ancestry-informative markers (https://github.com/nievergeltlab/global_ancestry). PCs in European ancestry were calculated using the smartPCA algorithm in EIGENSTRAT. Linear mixed models for the UKB GWAS were fit using Bolt LMM 2.3.2.<br><br>MVP genotype data was phased using Eagle 2.4 (reference panel: 1KGPp3) and minimac4 (reference panel: 1KGPp3). Relatedness was estimated using KING. PCs were calculated in each HARE ancestry group using FlashPCA2, and GWAS was done in PLINK 1.9, using logistic regression.<br><br>Sample size weighted fixed effects meta-analyses were conducted in METAL. Determination of the chromosome 8p23.1 inversion genotype was accomplished using the R package invClust. Regional association plots were produced in LocusZoom 1.4 (LD calculations based on 1KGPp3 data). Functional annotation of GWAS results was done with the FUMA pipeline v1.3.7, including the MAGMA tool for gene-based, gene-pathway, and tissue enrichment analyses. Polygenic functionally-informed fine-mapping (Polyfun) was performed with Polyfun, and tinnitus risk loci fine-mapping was done using SUSIE. PRS-CS was used to determine the posterior effect size of SNPs. Data processing for Jean et al. gene expression data was performed with the R package Seurat 4.9.9.9058. LD score regression and cross-trait genetic correlations were performed using the Complex Trait Genetics Virtual Lab (https://vl.genoma.io/). Univariate MiXeR 1.3 was used to estimate genetic architecture, and bivariate MiXeR was used to calculate phenotype-specific polygenicity and shared polygenicity between phenotypes. Case-case GWAS was performed with the R package CC-GWAS. Genomic structural equation modeling was performed with GenomicSEM.

April 2023

The codes for the analysis are available on Github (https://doi.org/10.5281/zenodo.10093735; https://github.com/nievergeltlab/Tinnitus).

For manuscripts utilizing custom algorithms or software that are central to the research but not yet described in published literature, software must be made available to editors and reviewers. We strongly encourage code deposition in a community repository (e.g. GitHub). See the Nature Portfolio guidelines for submitting code & software for further information.

Data

Policy information about availability of data

All manuscripts must include a data availability statement. This statement should provide the following information, where applicable:

- Accession codes, unique identifiers, or web links for publicly available datasets
- A description of any restrictions on data availability
- For clinical datasets or third party data, please ensure that the statement adheres to our policy

Summary statistics for the UKB GWAS used in this study have been deposited on figshare repository (https://doi.org/10.6084/m9.figshare.24121281.v1).113 Summary statistics are publicly accessible on figshare; raw data are protected and are not available due to privacy reasons. Summary statistics for MVP analyses will be deposited upon publication on dbGaP under accession number phs001672 (https://www.ncbi.nlm.nih.gov/projects/gap/cgi-bin/study.cgi?study_id=phs001672.v11.p1). MVP summary data access can be obtained by submitting a data access request through dbGaP; raw data are protected and are not available due to privacy reasons. The dataset from Hoa et al. was made available through correspondence with the authors, and the dataset from Jean et al. are available on the gEAR portal (https://umgear.org//index.html?share_id=9c42d685&gene_symbol_exact_match=1). The programs LocusZoom, Polyfun, and FUMA provide the reference panels and datasets used in the described analysis; drug-class and drug-set analyses were done using the Drug Gene Interaction Database DGIdb v4.2.0 (https://www.dgidb.org/downloads), Psychoactive Drug Screening Database Ki Database (https://pdsp.unc.edu/databases/kiDownload/), ChEMBL v27 (https://chembl.gitbook.io/chembl-interface-documentation/downloads), Target Central Resource Database v6.7.0 (http://juniper.health.unm.edu/tcrd/download/), and DSigDB v1.0 (https://dsigdb.tanlab.org/DSigDBv1.0/download.html).

Research involving human participants, their data, or biological material

Policy information about studies with human participants or human data. See also policy information about sex, gender (identity/presentation), and sexual orientation and race, ethnicity and racism.

Reporting on sex and gender	Sex was considered in the study design. Sex was determined from genetic data. The UKB sample was 54% female (N=80,018 men and 92,997 women). The UKB data, which has been previously published, reports sex-stratified analyses.<br><br>The MVP sample was approximately 7% (N=388,106 men and 35,804 women), and thus sex-stratified analyses within the MVP could not be performed with reasonable statistical power.<br><br>The joint analysis performed includes both sexes, because near or at unity genetic correlation between the sexes implies that such analyses can be conducted without loss of generality of results to either sex. The total numbers of subjects, stratified by sex, study, and case status, are listed in Supplementary Data 1 Table 1.
Reporting on race, ethnicity, or other socially relevant groupings	Biogeographical ancestry was considered in the study design.<br><br>Ancestry was determined for the UKB through the SNPweights program using ancestry informative genetic markers (https://github.com/nievergeltlab/global_ancestry). UKB analysis included individuals of European ancestry, due to insufficient sample sizes in other ancestries.<br><br>HARE (harmonized ancestry and race/ethnicity) estimates were used to defined subjects as non-Hispanic white (corresponding to European ancestry), non-Hispanic black (corresponding to African ancestry), and Hispanics (corresponding to Latinx ancestry). GWAS was perfomed separately in each HARE group and used PCs calculated in each ancestry separately.<br><br>Analyses were stratified by ancestry (N=481,874 European, 82,343 African, 32,688 Hispanic). Cross ancestry meta analysis was conducted using an inverse variance weighted meta analysis.
Population characteristics	The average age of participants was approximately 59 years old in the UKB and 64 years old in the MVP. UKB participants were genotyped on Affymetrix Axiom or UK BiLEVE Axiom arrays, and MVP participants were genotyped on the Affymetrix Axiom Biobank array. 19% of UKB subjects reported tinnitus at least some of the time, and 12% reported tinnitus in the past but not currently. 26% of MVP subjects had an ICD diagnosis of tinnitus, and 32% self-reported having tinnitus; 36% of MVP subjects either had an ICD diagnosis or self-report of tinnitus.
Recruitment	The UKB recruited 503,317 adults between 2006-2010 out of a population with 9.2 million men and women (5.45% recruitment rate) registered in the UK National Health Service within England, Scotland, and Wales who were randomly invited to participate.<br><br>The MVP made available data from 462,335 participants recruited since 2011 in version 18_2 (released March 27, 2019), which contains MVP enrollees through January 9, 2019 with MVP survey data through January 18, 2019 and ICD data from the Corporate Data Warehouse through September 30, 2018. Participants filled out a basic health question survey, and information including ICD diagnostic codes has been linked to individuals, de-identified health records.<br><br>Sample selection biases may have been present. For the MVP, successful induction into the military may imply differences from civilian populations. The UKBmay be unique in that participants have generally higher incomes and therefore may not represent the average person from the populations they are drawn from. These biases may generally affect external validity of results, however, we note that we have examined data and found that the general genetic signal we identify broadly

replicates across them.

Ethics oversight	The study was approved by the University of California San Diego and the VA CIRB and VASDHS R&D Institutional Review Boards.

Note that full information on the approval of the study protocol must also be provided in the manuscript.

Field-specific reporting

Please select the one below that is the best fit for your research. If you are not sure, read the appropriate sections before making your selection.

[X] Life sciences [ ] Behavioural & social sciences [ ] Ecological, evolutionary & environmental sciences

For a reference copy of the document with all sections, see nature.com/documents/nr-reporting-summary-flat.pdf

Life sciences study design

All studies must disclose on these points even when the disclosure is negative.

Sample size	Sample size was not predetermined but instead reflects our best effort to aggregate large studies with genome-wide genotype data and phenotyping of tinnitus. This study includes the Million Veterans Program and UK Biobank, and our knowledge represents the largest genome-wide study of tinnitus to date. Based on the available data, we have made efforts to maximize the use of the genotyped samples. This includes developing the infrastructure and appropriate statistical modeling to include unrelated/related samples and cross-ancestral analysis of African, European, and Latinx ancestry individuals. We have also performed power analysis for the current genome-wide study. For instance, we estimate that the full discovery meta-analysis has >80% power to detect variants associated with PTSD with true odds ratios >= 1.1 and minor allele frequency > 0.2. This power and sample size are consistent with successful GWAS of many other highly polygenic traits.
Data exclusions	Data exclusions were performed based on (a) failure of pre-determined data quality control criteria and (b) planned phenotype exclusions to ensure valid case/control criteria. For quality control, individuals were excluded if they were observed to have low genotyping quality (detailed in methods). Ancestries other than African, European, and Latinx ancestries were excluded due to insufficient sample size for a meaningful analysis in the currently available data. For phenotype-based exclusions, we omit individuals lacking phenotype information for tinnitus. The metrics used as exclusion criteria were established prior to analyses, but some thresholds used for exclusion (e.g. cutoffs from ancestry analysis to define ancestry strata) were evaluated during the QC process. All of the above exclusions were made in accordance with the planned study protocol and are detailed in the manuscript.
Replication	We attempted cross-ancestry replication of all genome-wide significant loci in the study. As described in the manuscript, direct replication was minimal. We note that lack of replication across ancestry groups may be due to lack of power in the replication samples or differing linkage disequilibrium patterns. For replication of a general tinnitus signal in the data and to indicate generalizability of the overall results across cohorts, polygenic risk score analyses and genetic correlations were used. In all instances, polygenic risk scores derived from subsets of this study successfully predicted tinnitus phenotypes in holdout data and significant genetic correlation was seen across different subsets of the data.
Randomization	Randomization of experimental groups was not applicable to this study. The experimental conditions are determined by each individual's genetics, which are fixed at conception. Conceptually, this reflects a randomization of the alleles inherited from each individual's parents (i.e., Mendelian randomization), but it does not involve randomization of experimental conditions in a classical sense. Our study assesses the observed association between that natural randomization of genotype and the ascertained phenotype of PTSD.
Blinding	Blinding is not relevant to the current study. Samples were not allocated to different conditions by the researches, and the phenotype ascertainment process is fully separate from the genotyping process.

Reporting for specific materials, systems and methods

We require information from authors about some types of materials, experimental systems and methods used in many studies. Here, indicate whether each material, system or method listed is relevant to your study. If you are not sure if a list item applies to your research, read the appropriate section before selecting a response.

Materials & experimental systems

n/a	Involved in the study	
[X]	[ ]	Antibodies
[X]	[ ]	Eukaryotic cell lines
[X]	[ ]	Palaeontology and archaeology
[X]	[ ]	Animals and other organisms
[X]	[ ]	Clinical data
[X]	[ ]	Dual use research of concern
[X]	[ ]	Plants

Methods

n/a	Involved in the study	
[X]	[ ]	ChIP-seq
[X]	[ ]	Flow cytometry
[X]	[ ]	MRI-based neuroimaging

Plants

Seed stocks	*Report on the source of all seed stocks or other plant material used. If applicable, state the seed stock centre and catalogue number. If plant specimens were collected from the field, describe the collection location, date and sampling procedures.*
Novel plant genotypes	*Describe the methods by which all novel plant genotypes were produced. This includes those generated by transgenic approaches, gene editing, chemical/radiation-based mutagenesis and hybridization. For transgenic lines, describe the transformation method, the number of independent lines analyzed and the generation upon which experiments were performed. For gene-edited lines, describe the editor used, the endogenous sequence targeted for editing, the targeting guide RNA sequence (if applicable) and how the editor was applied.*
Authentication	*Describe any authentication procedures for each seed stock used or novel genotype generated. Describe any experiments used to assess the effect of a mutation and, where applicable, how potential secondary effects (e.g. second site T-DNA insertions, mosiacism, off-target gene editing) were examined.*
